# The extracellular-regulated protein kinase 5 (ERK5) enhances metastatic burden in triple-negative breast cancer through focal adhesion protein kinase (FAK)-mediated regulation of cell adhesion

**DOI:** 10.1038/s41388-021-01798-2

**Published:** 2021-05-12

**Authors:** Qiuping Xu, Jingwei Zhang, Brian A. Telfer, Hao Zhang, Nisha Ali, Fuhui Chen, Blanca Risa, Adam J. Pearson, Wei Zhang, Katherine G. Finegan, Ahmet Ucar, Emanuele Giurisato, Cathy Tournier

**Affiliations:** 1 Division of Cancer Sciences, School of Medical Sciences, Faculty of Biology, Medicine and Health, University of Manchester, Manchester, UK; 2 Division of Pharmacy and Optometry, School of Health Sciences, Faculty of Biology, Medicine and Health, University of Manchester, Manchester, UK; 3 State Key Laboratory of Biocontrol, School of Life Sciences, Sun Yat-sen University, Guangzhou, China; 4 Manchester University NHS FT, Wythenshawe hospital, Manchester, UK; 5 Department of Biotechnology Chemistry and Pharmacy, University of Siena, Siena, Italy

**Keywords:** Breast cancer, Metastasis, Focal adhesion, Extracellular signalling molecules, Phosphorylation

## Abstract

There is overwhelming clinical evidence that the extracellular-regulated protein kinase 5 (ERK5) is significantly dysregulated in human breast cancer. However, there is no definite understanding of the requirement of ERK5 in tumor growth and metastasis due to very limited characterization of the pathway in disease models. In this study, we report that a high level of ERK5 is a predictive marker of metastatic breast cancer. Mechanistically, our in vitro data revealed that ERK5 was critical for maintaining the invasive capability of triple-negative breast cancer (TNBC) cells through focal adhesion protein kinase (FAK) activation. Specifically, we found that phosphorylation of FAK at Tyr397 was controlled by a kinase-independent function of ERK5. Accordingly, silencing ERK5 in mammary tumor grafts impaired FAK phosphorylation at Tyr397 and suppressed TNBC cell metastasis to the lung without preventing tumor growth. Collectively, these results establish a functional relationship between ERK5 and FAK signaling in promoting malignancy. Thus, targeting the oncogenic ERK5-FAK axis represents a promising therapeutic strategy for breast cancer exhibiting aggressive clinical behavior.

## Introduction

The overall survival of breast cancer patients has increased quite remarkably over the past few decades as a consequence of the introduction of early detection programs, but also due to the utilization of improved treatment protocols based on molecular and genomic diagnostics [1]. In particular, patterns of expression of the estrogen receptor (ER), progesterone receptor (PR) and human epidermal growth factor receptor 2 (HER2) constitute effective predictive markers for potential responses to endocrine treatment, the humanized monoclonal anti-HER2 antibody trastuzumab (Herceptin®) and the small protein tyrosine kinase inhibitor lapatinib [2, 3]. In spite of these marked therapeutic advances, the most aggressive basal-like tumors, which largely overlap with the triple ER, PR and HER2 negative subtype of breast cancer (TNBC), continue to present a significant clinical challenge due to the limited availability of treatment options [2, 3]. Notably, regardless of the combination chemotherapy regimen given, many responses in the metastatic setting are short, with a median overall survival being of less than 2 years [3]. Therefore, the identification of relevant biomarkers has become of paramount importance for guiding the future development of efficacious targeted therapies to improve TNBC patient outcome.

Signal transduction via mitogen-activated protein kinases (MAPK) are frequently dysregulated in many types of cancer. In particular, there is clinical evidence that overexpression and hyperphosphorylation of the extracellular-regulated protein kinase 5 (ERK5) are associated with overall poor survival rates of breast cancer patients and resistance to chemotherapy [4–7]. Accordingly, mammary graft studies, using human (MDA-MB-231) or murine (4T1) breast cancer cell lines that mirror the molecular subtype of TNBC, demonstrated that ERK5 knockdown suppressed metastasis [8–10]. Interestingly, mice transplanted with MDA-MB-231 cells in which ERK5 was silenced exhibited a decreased number of circulating tumor cells in the blood compared with those transplanted with wild type MDA-MB-231 cells [9]. Collectively these observations in murine models added weight to the argument, from the analysis of patient tumor samples, that ERK5 targeting represented a valid strategy against metastatic breast cancer.

In contrast, there is no clarity in understanding the mechanism by which ERK5 influences metastasis-related tumor cell function. For example, ERK5 could function via rearranging the epithelial phenotype of breast cancer cells to acquire migratory and invasive characteristics [9–11]. This has been disputed by other studies which suggested that ERK5 attenuated breast cancer cell motility and suppressed mammary tumor metastasis through negative regulation of mesenchymal markers [12, 13]. Additionally, ERK5 was found to be a target of the focal adhesion protein kinase (FAK) in breast cancer cell adhesion signaling [14]. However, a functional relationship between ERK5 and FAK signaling in the pathogenesis of metastatic breast cancer has never been fully investigated in vivo. Furthermore, the precise mechanistic interaction between ERK5 and FAK remains unclear. In particular, while ERK5 was initially found to be a target of FAK [14], other studies demonstrated that ERK5 promoted breast cancer cell proliferation [15], adhesion-dependent survival [16], and melanoma cell invasiveness [17] through FAK phosphorylation at Ser910.

In this study, we have utilized the triple-negative MDA-MB-231 breast cancer line and selective short hairpin RNA (shRNA) knockdown strategy to assess the cellular function under the control of ERK5 that influences the course of breast tumor progression and metastasis. The demonstration that ERK5 was required for FAK phosphorylation at Tyr397 and breast cancer metastasis identifies a previously untargeted signaling axis implicated in tumor cell migration for distant metastasis formation.

## Results

### High ERK5 expression serves as a potentially important prognostic indicator of breast cancer malignancy and poor prognosis

**Fig. 1:**
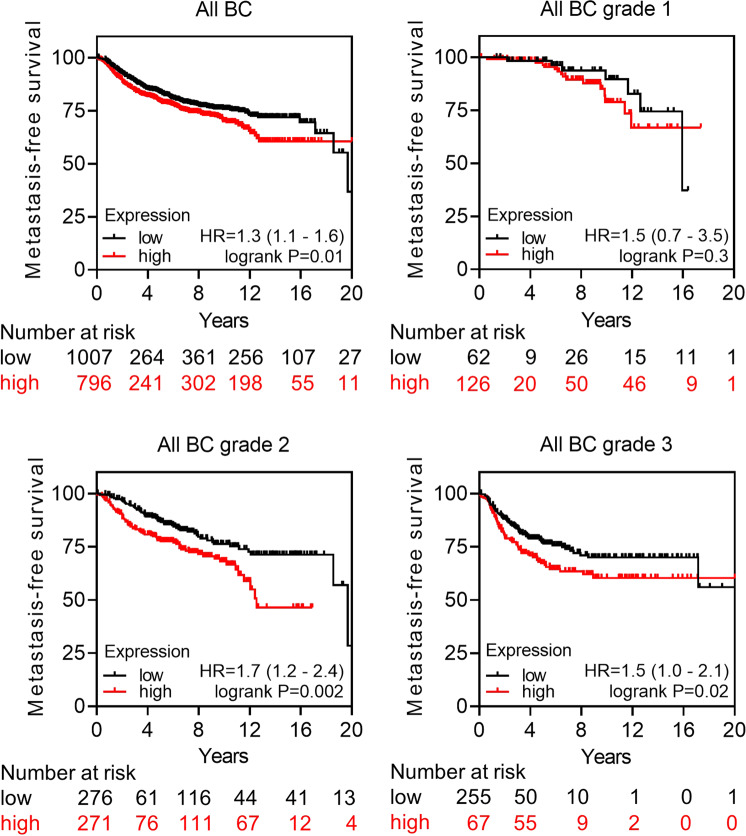
ERK5 expression strongly associates with metastatic occurrence in breast cancer. The relationship between the level of ERK5 expression and distant metastasis-free survival of breast cancer (BC) patients was assessed by Kaplan–Meier plotter. Independent cohorts of patients with early (grade 1) or late (grades 2 and 3) stage disease status were analyzed. Samples were divided into two groups with high (red) and low (black) expression of ERK5. Hazard ratios (HR) and logrank *P* values are shown.

**Fig. 2:**
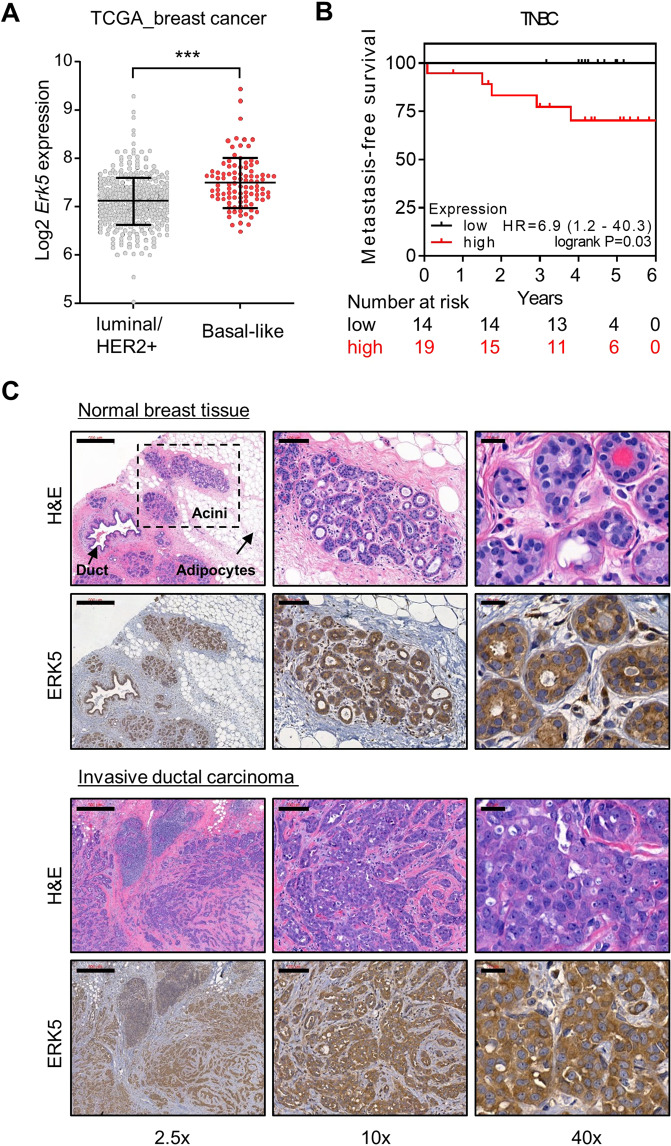
ERK5 is overexpressed in TNBC and its overexpression strongly correlates with poor prognosis. **A** The level of *Erk5* transcript was analyzed in 416 luminal/HER2+ tumors and 98 TNBC from TCGA dataset. Black lines in each group indicate median with interquartile range. **B** Kaplan–Meier analysis of distant metastasis-free survival of TNBC patients. Samples were divided into two groups with high (red) and low (black) expression of ERK5. Hazard ratio (HR) and logrank *P* values are shown. **C** A biopsy of human invasive ductal carcinoma and adjacent normal breast tissue (patient #481) was stained with H&E or with a specific antibody to ERK5 (brown). Scale bars: (2.5x) 500 μm, (10x) 100 μm, (40x) 20 μm.

Initially, we investigated the clinical significance of ERK5 in breast cancer using the Kaplan–Meier Plotter database tool (kmplot.com). We found that the level of ERK5 expression inversely correlated with distant metastasis-free survival across all subtypes (hazard ratio = 1.3, *p* value = 0.01; Fig. 1). The association of ERK5 expression with the metastatic spread of the disease was statistically significant in later stage breast cancer (all hazard ratios ≥1.5, *p* value = 0.3 for grade 1 compared with <0.02 for grades 2 and 3). In parallel, the analysis of a large transcriptomic dataset of breast tumors from The Cancer Genome Atlas (TCGA) [18] indicated that basal-like tumors exhibited the highest expression level of *Erk5* transcript among all subtypes, i.e., luminal and HER2-positive subgroups (Fig. 2A). Triple-negative molecular subtyping is routinely utilized in the clinic as a surrogate classification for aggressive basal-like tumors lacking ER/PR and HER2 expression. We found a robust inverse correlation between the level of ERK5 expression and metastasis-free survival of TNBC patients (hazard ratio = 6.9, *p* value = 0.03; Fig. 2B).

We subsequently analyzed by immunohistochemistry biopsies of human triple-negative ductal carcinoma and adjacent normal breast tissue, as well as lung metastatic sites. In normal breast and lung tissues, ERK5 was specifically detected in acini, ducts and alveoli (Fig. 2C and Supplementary Fig. S1). Higher magnifications clearly demonstrated a predominant ERK5 staining in the cytoplasm of mammary acinar cells. Unlike normal tissue, high-grade invasive ductal carcinoma appeared highly disorganized. The notable absence of tubule formation was associated with the presence of numerous pleomorphic cells exhibiting increased mitotic activity (Fig. 2C and Supplementary Fig. S1). Nonetheless, ERK5 remained predominantly cytoplasmic in cancerous cells with some evidence of perinuclear staining, suggesting that ERK5 was not constitutively activated in triple-negative ductal carcinoma (Fig. 2C and Supplementary Fig. S1). A similar pattern of ERK5 staining was detected in metastatic breast cancer cells from lung biopsies (Supplementary Fig. S1).

Moreover, we confirmed that the protein abundance of ERK5 was higher in TNBC cell lines (MDA-MB-231 and MDA-MB-468) compared with HER2-positive breast cancer cells (BT474, SK-BR-3 and MDA-MB-453) (Supplementary Fig. S2A). Remarkably, ERK5 expressed in TNBC cells did not exhibit the typical mobility shift by SDS-polyacrylamide gel electrophoresis (SDS-PAGE) characteristic of ERK5 activation by hyperphosphorylation downstream of HER2 stimulation [19]. The absence of ERK5 hyperphosphorylation in TNBC cell lines was consistent with the lack of nuclear ERK5 staining in sections of human triple-negative breast carcinoma.

### ERK5 is required for breast cancer cell invasion and the suppression of E-cadherin expression

**Fig. 3:**
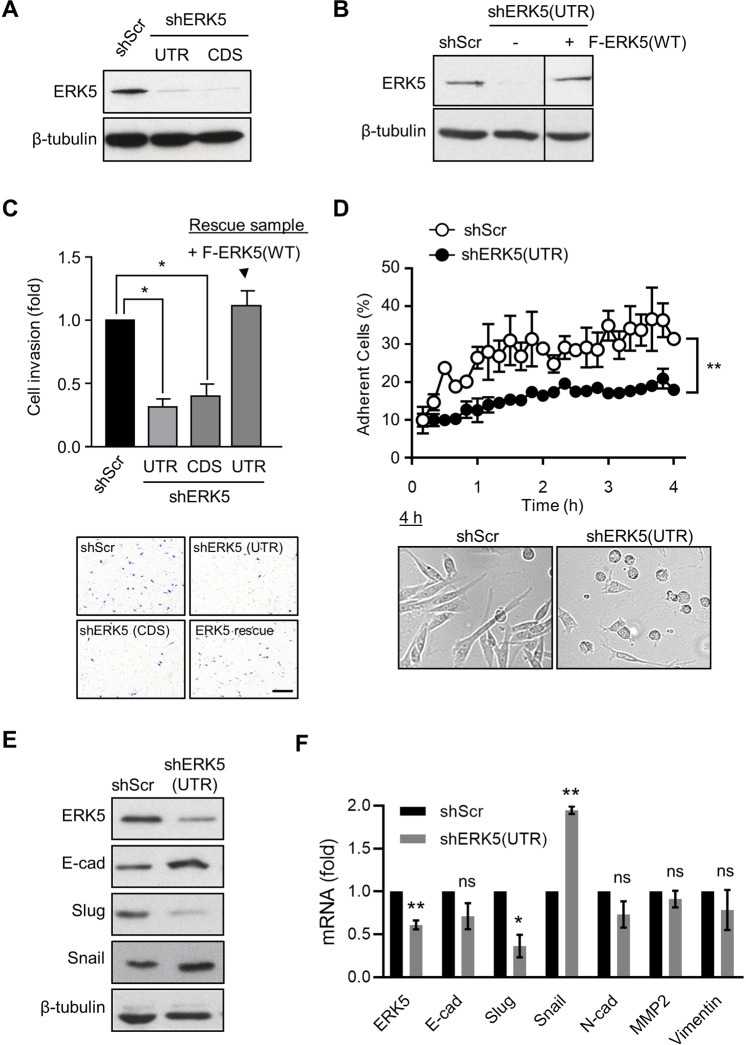
ERK5 silencing suppresses breast cancer cell invasion. **A**, **B** Immunoblot analysis confirmed that shERK5(UTR) and shERK5(CDS) effectively downregulated ERK5 expression in MDA-MB-231 cells. Ectopic expression of ERK5 [+ F-ERK5(WT)] at a level similar to that of the endogenous protein was achieved by incubating shERK5(UTR)-expressing cells carrying an inducible F-ERK5(WT) construct with 10 ng/ml doxycycline (+) for 24 h. **C** ERK5 knockdown suppressed MDA-MB-231 cell invasion in FBS-free medium through Matrigel. The bar graph indicates the mean ± SD (*N* = 3). Representative pictures of the filters are shown. Scale bar: 200 μm. **D** Quantitative time course analysis of MDA-MB-231 cell adhesion on Matrigel by IncuCyte® live-cell imaging system. Representative pictures of cells taken 4 h after seeding in Matrigel-coated plates are shown. The data correspond to the mean ± SD (*N* = 3). **E** Lysates were obtained from cells cultured in FBS-free Matrigel-coated plates for 12 h and analyzed by immunoblot. The images are representative of three biological repeats. **F** Alternatively, transcript levels were measured by quantitative real-time PCR. Results are presented as fold change ± SD. The graph is representative of three independent biological repeats performed in duplicate.

To gain further insights into the oncogenic function of ERK5 in breast cancer, we engineered stable ERK5 knockdown in MDA-MB-231 cells using two distinct shRNAs targeting the 3′UTR or the coding sequence (CDS) of ERK5. MDA-MB-231 cells transduced with non-target scrambled (Scr) shRNA were utilized as controls. The efficacy of both shRNA to silence ERK5 expression was demonstrated by immunoblot analysis (Fig. 3A, B).

In contrast to ERK5 inhibition by XMD8–92 [20], ERK5 knockdown did not prevent the growth of MDA-MB-231 cells (Supplementary Fig. S2B). Instead, we found that ERK5 silencing significantly impaired breast cancer cell invasion through the Matrigel under fetal bovine serum (FBS)-free condition or in response to EGF treatment, but not following FBS stimulation (Fig. 3C and Supplementary Fig. S2C). Importantly. we confirmed that the invasive capability of shERK5(UTR)-expressing MDA-MB-231 cells was restored following doxycycline-induced expression of FLAG-tagged (F) ERK5 wild type (WT) at a level similar to that of the endogenous protein (Fig. 3B, C). Additionally, real-time quantitative IncuCyte® live cell imaging revealed that silencing ERK5 reduced by around two-fold the kinetic of breast cancer cell adhesion to Matrigel-coated plates, independently of increased cell death up until 12 h after seeding (Fig. 3D and Video 1). Adherent shERK5(UTR)-expressing MDA-MB-231 cells detected at the end of the experiment exhibited a morphology similar to that of control shScr cell line (Video 1).

The reduced invasive capability of MDA-MB-231 cells caused by ERK5 silencing correlated with increased expression of the epithelial marker E-cadherin (Fig. 3E). Although the level of the *E-cadherin* transcript was not significantly altered, this coincided with a marked reduction in the expression of Slug, a transcriptional repressor of E-cadherin involved in EMT (Fig. 3E, F). Conversely, protein and RNA levels of Snail, another important transcriptional repressor of E-cadherin, were increased in shERK5(UTR)- compared with shScr-expressing MDA-MB-231 cells, while the transcript level of other mesenchymal markers (e.g., N-cadherin, MMP2, Vimentin) was unchanged (Fig. 3F).

### FAK is a potentially important regulator of breast cancer metastasis controlled by a kinase-independent function of ERK5

To further explore the underlying mechanism of ERK5-mediated breast cancer cell invasion, we tested the association between ERK5 and FAK, a non-receptor protein tyrosine kinase that plays a prominent role in cell spreading and migration, and the initiation of various malignancies [21]. The best-characterized mechanism that promotes FAK activation upon cell interactions with the extracellular matrix involves FAK dimerization and subsequent autophosphorylation at Tyr397 [22]. Interestingly, ERK5-mediated FAK phosphorylation at Ser910 during melanoma cell migration coincided with decreased phosphorylation at Tyr397 [17].

**Fig. 4:**
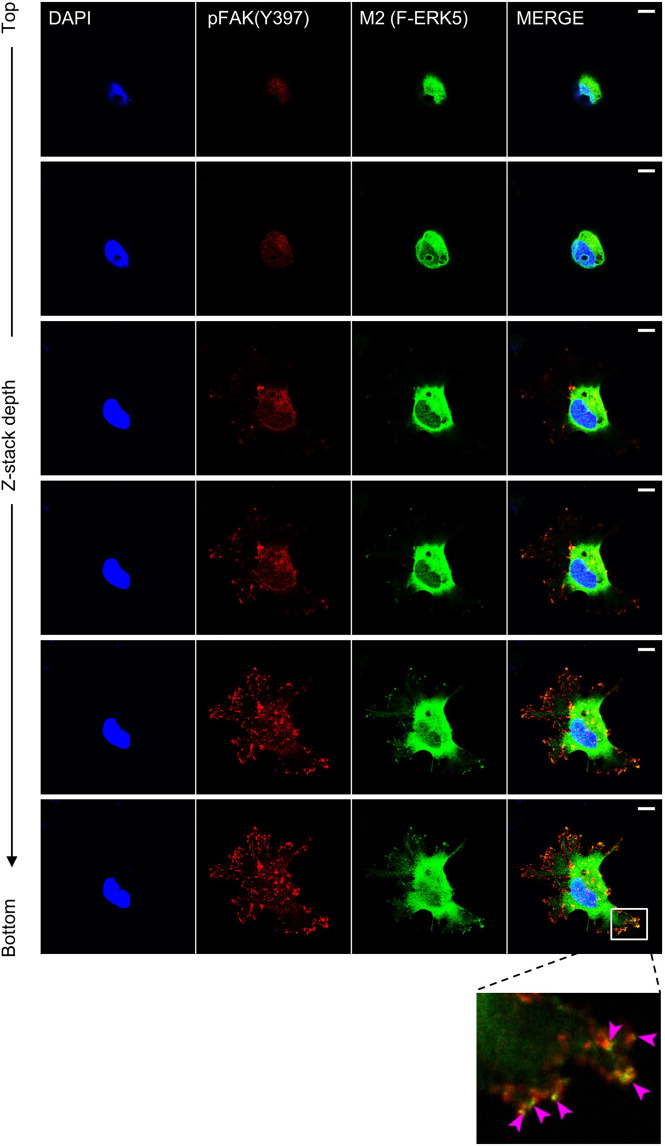
ERK5 co-localizes with p-FAK in focal adhesions. iRFP720^+^ MDA-MB-231 cells carrying shERK5(UTR) were incubated for 24 h with 2 μg/ml doxycycline to induce F-ERK5(WT) expression, prior to being seeded on glass bottom dish coated with Matrigel and cultured for 6 h in FBS-containing medium. Ectopically expressed F-ERK5 and endogenous FAK were visualized by confocal microscopy using specific antibodies to the FLAG epitope (M2, green) and to p-FAK(Y397) (red). Nuclei were detected with DAPI (blue). Images at different Z positions are shown. Scale bars: 10 μm. The inset shows a higher magnification of focal adhesions where F-ERK5 and p-FAK(Y397) co-localized. Violet arrows highlight yellow staining indicative of F-ERK5/p-FAK(Y397) co-localization.

**Fig. 5:**
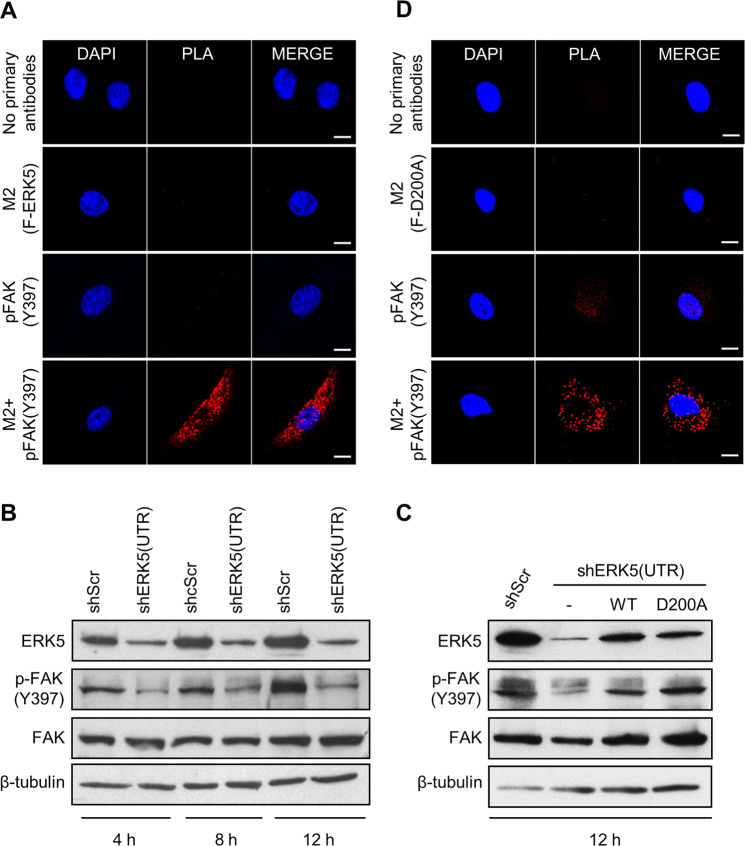
ERK5 interacts with FAK signaling. iRFP720^+^ MDA-MB-231 cells carrying shERK5(UTR) were incubated for 24 h with 2 μg/ml doxycycline to induce F-ERK5(WT) (**A**) or F-ERK5(D200A) (**D**) expression. Cells were subsequently seeded on Matrigel-coated glass bottom plate for 6 h prior to being fixed and incubated with antibodies to the FLAG epitope (M2) and p-FAK(Y397). Negative controls included incubation with each primary antibody separately and no primary antibodies. In situ detection of ERK5-FAK complexes was performed with oligonucleotide-labeled secondary antibodies according to the Duolink^®^ PLA fluorescence protocol (Sigma). Images were acquired by fluorescence microscopy. Scale bars: 10 μm. **B**, **C** Lysates were obtained from cells cultured in FBS-free Matrigel-coated plates for the indicated times. In panel **C**, iRFP720^+^ cells expressing shERK5(UTR) were incubated with 10 ng/ml doxycycline for 24 h to induce F-ERK5(WT) or F-ERK5(D200A) expression, prior to being seeded. MDA-MB-231 cells expressing shScr control or transduced with an empty pCHD plasmid (−) were utilized as controls. Similar results were obtained in two independent experiments.

Here, we found that a proportion of ectopically expressed F-ERK5(WT) in shERK5(UTR)-expressing MDA-MB-231 cells co-localized with phosphorylated FAK at Tyr397 in focal adhesion sites (Fig. 4). Three-dimensional confocal microscopy clearly showed dual staining of F-ERK5(WT) and phospho (p)-FAK(Y397) at cell surface attachment points with the Matrigel (Video 2). We subsequently confirmed by in situ proximity ligation assay (PLA) that F-ERK5(WT) could be detected in very close proximity with FAK, indicative of the existence of an ERK5-FAK complex (Fig. 5A). The specificity of this interaction was supported by evidence that a C-terminal truncated ERK5 mutant [F-ERK5-ΔC(1–575)] displaying a predominant nuclear localization [23] did not associate with FAK (Supplementary Fig. S3A).

In parallel, we discovered that ERK5 silencing in breast cancer cells inhibited FAK phosphorylation at Tyr397 (Fig. 5B). The demonstration that doxycycline-induced expression of F-ERK5(WT) restored normal level of FAK phosphorylation in shERK5(UTR)-expressing MDA-MB-231 cells confirmed that ERK5 was required for FAK activation in response to signals from the extracellular matrix (Fig. 5C). Remarkably, normal FAK phosphorylation in shERK5(UTR)-expressing MDA-MB-231 cells was also achieved following induced expression of F-ERK5(D200A), a kinase dead mutant of ERK5 unable to bind ATP [24] (Fig. 5C). We confirmed that JWG-045, a novel pharmacological inhibitor of ERK5 exhibiting significantly reduced affinity for BRD4 compared with XMD8–92 [25, 26], did not suppress FAK phosphorylation at Tyr397 in MDA-MB-231 cells (Supplementary Fig. S3B, C). Interestingly, neither ERK5 silencing nor incubation with JWG-045 affected the level of FAK phosphorylation at Ser910 (Supplementary Fig. S3C). Collectively, these results demonstrated that ERK5 controlled the post-translational modification of FAK at Tyr397, independently of its catalytic activity. Accordingly, F-ERK5(D200A) was able to associate with FAK (Fig. 5D) and also to rescue the invasion defect caused by ERK5 silencing (Supplementary Fig. S3D, E).

The functional importance of FAK activation downstream of ERK5 was demonstrated by evidence that overexpression of tyrosine phosphorylated FAK at the cell membrane [myristoylated (myr)FAK; [27] rescued the invasion defect caused by ERK5 silencing (Supplementary Fig. S3D, E). Additionally, we confirmed the clinical significance of FAK in TNBC (Supplementary Fig. S4A–C). Consistent with evidence that FAK signaling inversely correlated with metastasis-free survival, inhibiting FAK activity by incubating MDA-MB-231 cells with PND1186 [28] significantly decreased their ability to invade through the Matrigel (Supplementary Fig. S4D). However, PND1186 did not affect the level of ERK5 expression and ERK1/2 phosphorylation (Supplementary Fig. S4E). Likewise, FAK inhibition did not affect the expression of E-cadherin, indicating that FAK was not involved in maintaining the mesenchymal phenotype of MDA-MB-231 cells downstream of ERK5 (Supplementary Fig. S4E).

### ERK5 is a critical component of malignant progression of breast cancer through FAK phosphorylation

To confirm the requirement of the ERK5-FAK axis in promoting breast cancer metastasis, we performed tumorigenesis assays in nude mice. First, we created a novel stable MDA-MB-231 cell line to permit doxycycline-inducible ERK5 knockdown in vivo (Supplementary Fig. S5A). MDA-MB-231 cells carrying the shERK5i-Luc2=tdT construct were transplanted into the mammary fat pad of athymic female CD1 mice to allow orthotopic tumor formation. Animals exhibiting small tumors (between 10 and 80 mm^3^ at day 20) were subsequently fed with a doxycycline containing diet (dox+) to silence ERK5 in breast cancer cells, or with a standard doxycycline-free (dox−) control diet. The presence of doxycycline in the serum of dox+ mice was confirmed by using mass-spectrometry analysis of terminal cardiac blood puncture samples (Supplementary Fig. S5B).

**Fig. 6:**
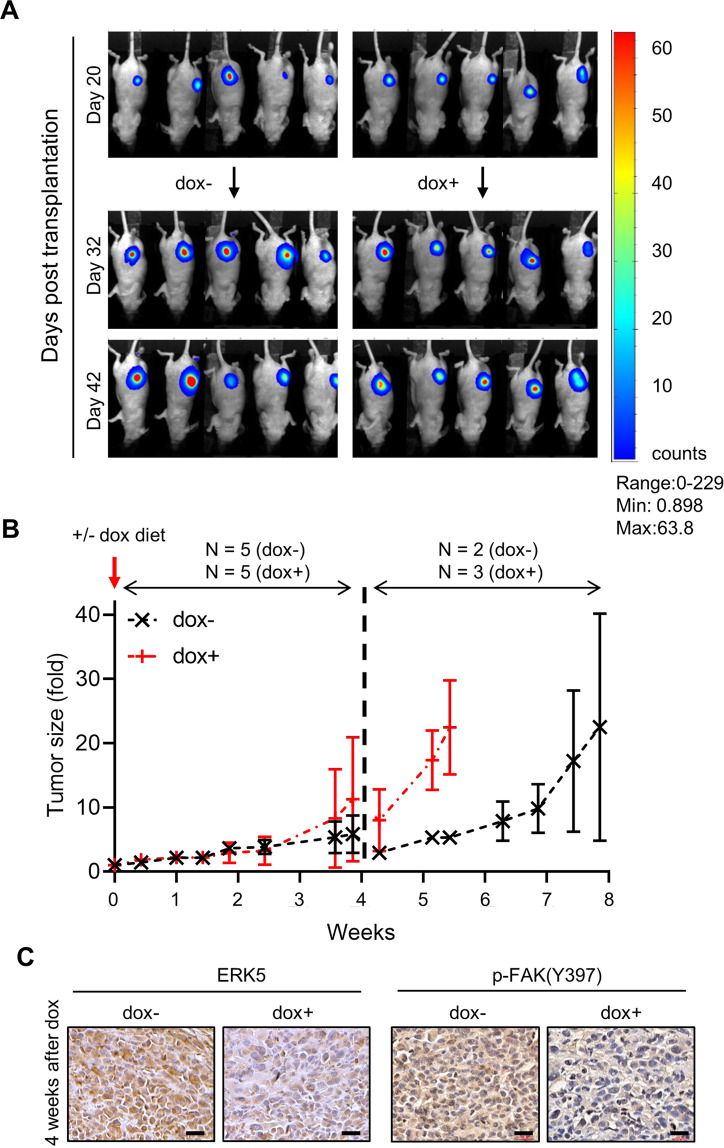
Induced ERK5 silencing accelerates tumor growth and suppresses FAK phosphorylation in mammary tumor grafts. MDA-MB-231 cells carrying shERK5i were orthotopically transplanted into the mammary fad pad of CD1 nude mice. **A** Representative bioluminescence images of mammary tumors just before (day 20) and after mice were fed with doxycycline for 12 and 22 days. **B** Tumors were measured on average twice a week for the duration of the experiment. The data presented as fold increase in the volume of tumor size after the introduction of the dox diet at day 20 correspond to the mean ± SD. After 4 weeks, animals in the dox− and dox+ cohorts exhibiting large tumors or a change in normal behavior were humanely culled. Total *N* number of animals analyzed per condition over the duration of the experiment is indicated. **C** Tumors were collected 4 weeks after exposure to doxycycline and analyzed by immunohistochemistry with a specific antibody to ERK5 or to p-FAK(Y397). Scale bars: (40x) 20 μm.

Initially, ERK5 silencing appeared to cause a slight decrease in tumor growth (Fig. 6A). However, no significant difference in tumor size was measured until the absence of ERK5 begun to noticeably accelerate the growth of orthotopic mammary grafts in mice exposed to doxycycline for 3.5 weeks, corresponding to 44 days post transplantation (Fig. 6B). A similar impact of ERK5 silencing on tumor growth was observed in a preliminary small pilot experiment in which MDA-MB-231 cells carrying the shERK5i-Luc2=tdT construct were transplanted subcutaneously into the back of adult female CBA nude mice (Supplementary Fig. S6A, B). Immunostaining of harvested tumor sections confirmed efficient silencing of ERK5 in orthotopic mammary grafts excised from dox+ CD1 mice (Fig. 6C). Remarkably, the reduction in ERK5 expression correlated with decreased p-FAK(Tyr397) staining, consistent with the requirement of ERK5 for mediating FAK activation in vivo (Fig. 6C). Accordingly, human invasive ductal carcinoma biopsies exhibiting high ERK5 expression displayed high level of p-FAK(Tyr397) (Supplementary Fig. S7).

**Fig. 7:**
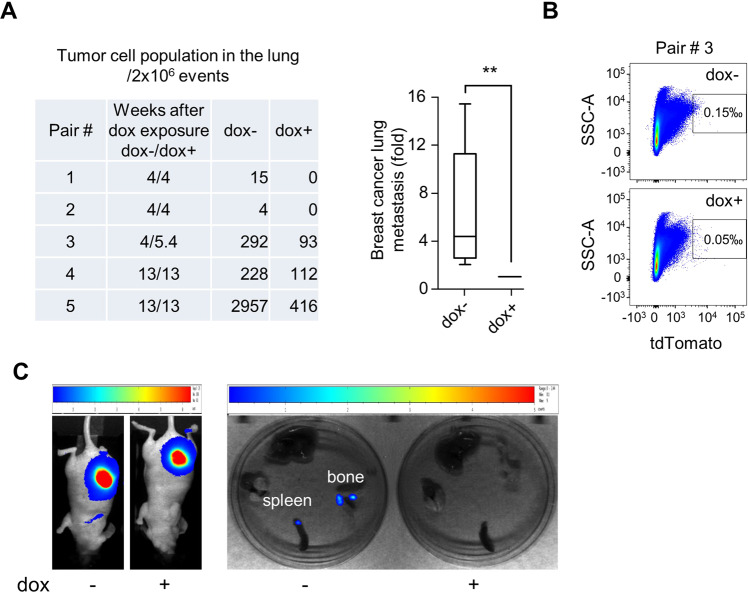
Induced ERK5 silencing suppresses breast cancer lung metastasis. Control (dox−) and doxycycline-fed (dox+) mice bearing mammary tumor grafts were paired according to similar tumor size. **A**, **B** Breast metastasis was detected post mortem by quantification of live (DAPI^−^) breast cancer cells (tdT^+^) in the lung. A representative flow cytometry analysis is shown in **B**. The graphical analysis of the data demonstrated that ERK5 knockdown significantly reduced metastatic burden. Ratio paired *t* test was applied for *p* value calculation, *p* ≤ 0.01 (**). Raw numbers utilized for the graph are presented in the table. **C** Breast metastasis to the spleen and bone were detected by bioluminescence imaging in one control (dox−) animal of a pair sacrificed at 4 weeks after dox exposure.

Mice cohorts were subsequently organized in pairs of dox+ and dox− animals according to similar size of tumor volumes (Fig. 7A). The presence of tdT+ metastatic breast cancer cells in the lungs of mice carrying orthotopic mammary tumor grafts was quantified post-mortem by flow cytometry (Fig. 7A, B). Consistent with the requirement of ERK5 for mediating breast cancer cell invasion in vitro, the absence of ERK5 notably suppressed breast cancer lung metastasis in vivo. The remaining low incidence of lung metastasis detected in the dox+ group was likely a consequence of residual ERK5 level in shERK5i-expressing MDA-MB-231 cells that would be expected in any silencing approach by means of inducible shRNA. Breast cancer cell metastasis to the spleen and the bone was also captured by bioluminescence imaging in one control animal among all recipient mice analyzed (Fig. 7C).

## Discussion

We have demonstrated for the first time that induced ERK5 silencing in small TNBC grafts accelerated the dynamic of tumor growth, suggesting that ERK5 could antagonize mitogenic signaling to maintain cancer cell survival in established tumors. This hypothesis contradicts previous reports that demonstrated that ERK5 knockdown did not affect the growth of primary triple-negative mammary tumors [8–10, 13]. Moreover, a recent publication provided new genetic evidence supporting the requirement of ERK5 in promoting tumorigenesis [29]. These conflicting findings highlight that continued efforts need to be made to resolve the ongoing controversy about the involvement of ERK5 in tumor growth during the early stage development of breast cancer. Nevertheless, ERK5 remains a clinically relevant therapeutic target in TNBC given its strong association with increased metastatic risk (8–10, 29, this study).

Disruption of the actin cytoskeleton by Src through ERK5-dependent transcription constituted the first evidence that ERK5 signaling altered the invasiveness of transformed cells [30, 31]. Here, we proposed that ERK5 maintained the invasive capability of breast cancer cells through FAK-mediated regulation of cell adhesion. Accordingly, we provided the first demonstration that decreased malignancy caused by ERK5 ablation associated with the loss of FAK phosphorylation at Tyr397 in mammary tumor grafts. Given the functional interaction between of Src and FAK in focal adhesion remodeling [32], this new finding emphasized a potentially important a role of ERK5 in metastatic mammary tumors with elevated c-Src kinase activity. An alternative mechanism underlying the metastatic effect of ERK5 involved the breast tumor kinase (Brk) downstream of the MET receptor [33, 34]. This might also be significant given that Brk is overexpressed in a majority of breast tumors, whilst undetectable in normal mammary gland [35].

Remarkably, ERK5-mediated FAK phosphorylation at Tyr397 in TNBC cells did not require ERK5 activity, suggesting that ERK5 could act as a scaffold protein to support cell invasion through FAK. Interestingly, ERK5 was mostly detected in the cytoplasmic compartment of cancerous cells in TNBC patients, consistent with the idea that ERK5 overexpression rather than hyperactivation is a significant feature of high-grade triple-negative invasive ductal carcinoma in humans. Conversely, ERK5 silencing did not affect FAK phosphorylation at Ser910, a site previously found to be phosphorylated by ERK1/2 [36], but also by ERK5 [17–19]. Increased FAK phosphorylation at Ser910 in response to ERK5 activation by MEK5 constituted an important mechanism for mediating melanoma lung metastasis [19]. Interestingly, high level of ERK5 and phosphorylated FAK at Ser910 in highly metastatic murine melanoma and human lung cancer cell lines coincided with low FAK phosphorylation at Tyr397 [19]. A dual function of ERK5 as a scaffold protein (our results) and an enzyme [17–19] might explain these apparent conflicting results.

In line with this idea, the estrogen receptor (ER)α was found to suppress breast cancer cell motility and invasion by recruiting ERK5 to the nucleus, thereby restricting the formation of an ERK5/cofilin complex in actin-rich regions of the cytoplasmic membrane [37]. Although the paper did not rigorously address whether or not ERK5 promoted the aggressiveness of ERα(−) breast cancer cells through modulating actin dynamics independently of its catalytic activity, estrogen-mediated transcription in ERα(+) breast cancer cells required ERK5 activation by MEK5 [38]. Interestingly, ectopic expression of MEK5 had been previously shown to promote a more malignant phenotype by repressing ERα expression and by promoting epithelial mesenchymal transition (EMT), a well-known reprograming process that confers metastatic properties to cancer cells [38–40].

Through genetic ablation and silencing approaches, ERK5 was also shown to be essential for maintaining mesenchymal features of MDA-MB-231 tumor grafts [9, 10, 29]. We confirmed that ERK5 suppressed the level of the epithelial marker E-cadherin downstream of Slug. This was not observed in PND1186-treated cells, suggesting that the impact of ERK5 silencing on E-cadherin expression was independent of impaired FAK signaling. Consistent with the CRISPR/Cas9 knock-out approach [29], ERK5 silencing increased the level of *Snail*. In contrast, we found no evidence that ERK5 altered the level of various mesenchymal markers, e.g., N-cadherin, vimentin, or expression of MMP2, a member of the MMP family known to potentiate cancer cell dissemination through degrading ECM. Moreover, the downregulation of ERK5 did not prevent adherent MDA-MB-231 cells from acquiring the typical stellate morphology characteristic of highly aggressive mesenchymal breast cancer model. These results led us to conclude that, although ERK5 might contribute to controlling the expression level of certain epithelial and mesenchymal markers, the pathological context under which inhibition of the ERK5 pathway is sufficient to reprogram an epithelial phenotype from an aggressive mesenchymal breast cancer model remains unknown.

In summary, we presented strong evidence that ERK5 overexpression was an important feature of TNBC metastasis through the regulation of cell adhesion via FAK. Based on our findings, we hypothesize that ERK5 might contribute to the dimerization of FAK as a prerequisite to FAK activation at focal adhesion sites, via its unique domain structure [23]. In particular, the C-terminal tail of ERK5 that comprises multiple phosphorylation sites, as well as proline-rich sequences, provides ideal binding motifs to serve a scaffolding function of ERK5 in TNBC [24, 41]. Thus, it will be critical to identify the membrane-associated ERK5 protein complex for understanding the requirement of ERK5 in FAK-mediated metastasis through controlling focal adhesion dynamics. Ultimately, these further investigations might help designing new treatment strategies for aggressive breast cancer based on disrupting the scaffolding function of ERK5, rather than inhibiting ERK5 activity.

## Materials and methods

### Lentivirus-mediated transfer plasmids

For constitutive ERK5 knockdown experiments in vitro, shRNA lentiviral transduction particles were purchased from Sigma-Aldrich to target the 3′UTR (#TRCN0000197264) or the CDS (#TRCN0000010275) of the *erk5* transcript. shScr lentiviral particles (#SHC016V) were utilized as controls. To generate doxycycline-dependent inducible ERK5shRNA, oligonucleotides were designed against the GACCCACCTTTCAGCCTTA sequence at position 5639–5657 in the 3′UTR of the *erk5* gene (Gene ID: 5598). Double-stranded oligonucleotides were subcloned using *Age*I and *EcoR*I in Tet-pLKO-puro-IRES-Luc2=tdT, a lentiviral transfer plasmid created by inserting a Luc2=tdT fragment (Addgene #32904) into the Tet-pLKO-puro vector (Addgene #21915). Sequences for shERK5i are available in Supplementary Table S1. To construct doxycycline-dependent inducible expression systems, F-ERK5(WT) or D200A mutant cDNAs were subcloned using *Pac*I and *Nhe*I into the bicistronic lentiviral expression vector pCHD-TRE3GS-MCS-EF1a-iRFP720 (a gift from Stuart Cain, University of Manchester, in which target cDNA expression is driven by the TRE3GS promoter and iRFP720 expression is driven by the EF-1α core promoter). Sequences for PCR amplification are available in Supplementary Table S1.

### Lentiviral infection of MDA-MB-231 cells

MDA-MB-231 cells were incubated for 24 h with viral particles at 1 to 2 MOI in the presence of 8 µg/ml polybrene. Infected cells with the pLKO recombinant plasmid were subsequently selected by incubation with 3 µg/ml puromycin until no live cells remained in the non-infected group (at least 3 days). Resistant colonies were pooled and expanded in puromycin-containing medium. Alternatively, MDA-MB-231 cells transduced with doxycycline-inducible F-ERK5(WT) or F-ERK5(D200A) delivering particles for 24 h were selected by FACS for iRFP720 fluorescence 2 days later.

### Transwell invasion assay and measurement of cell adhesion

For cell invasion, MDA-MB-231 cells were resuspended in FBS-free medium and seeded at a low density of 10,000 cells per well in the upper Boyden chamber of 24-well plates with 8-μm pore (Corning #353097) coated with Matrigel. The lower chamber was filled with culture medium without FBS. 16 h after seeding, the cells on the bottom layer surface were stained with 0.05% crystal violet dye and quantified under the microscope. Cell adhesion in Matrigel-coated plates was monitored by IncuCyte® live cell analysis. Four fields of view were set up for each well. The number of cells in each field was counted in real time, every 10 min. Adherent cells were distinguished from non-adherent cells based on a larger cell surface area. Triplicate data were used for statistical analysis.

### Mammary tumor grafts

Eighteen adult female CD1 nude mice were injected in the mammary fad pad (4th nipple orthotopic) with MDA-MB-231 cells carrying the shERK5(UTR-2)-Luc=tdT construct (2 × 10^6^ cells in 50 μl PBS mixed with 50 μl Matrigel). After small tumors were established, 9 mice received doxycycline in their diet, while 9 control animals were fed with a standard doxycycline-free diet. Tumor size was determined by caliper measurements of tumor length, width and depth and expressed as volume in mm^3^ (0.52 × length × width × depth). Four experimental mice in each cohort exhibited tumors that stopped growing few weeks after the introduction of the dox+ or dox− diets. Therefore, 5 animals per groups were utilized for statistical analysis to confirm the effect and rule out artefacts associated with biological variability. In parallel, tumor growth was analyzed by bioluminescence imaging. Mice received an intraperitoneal injection of 100 μl of luciferin (30 mg/ml) and imaged using a bioluminescent imaging system (PhotonImager Opitma, Biospace Lab, France). Images from dox− and dox+ group were captured under identical imaging conditions at identical times after luciferin injection.

### Analysis of mammary tumor lung metastasis by flow Cytometry

Metastasis was analyzed post mortem in the lung of mice bearing large mammary tumor grafts (around 700~800 mm^3^). In brief, lungs were collected, minced in a Petri dish on ice and sequentially digested in Hanks balanced salt solution (HBSS) containing collagenase (1.25 mg/ml) and hyaluronidase (125 μg/ml) for 30 min, trypsin-EDTA for 3 min, and dispase (5 mg/ml) and DNase (10 mg/ml) for 3 min. Neutralization with 1% FBS-containing HBSS was conducted between each enzymatic digestion, following centrifugation at 5000 rpm. The final cell suspension was filtered through a 40 μm cell strainer. Red blood cells were solubilized with red cell lysis buffer (Pharm Lyse, BD Biosciences) and the resulting suspension was filtered through a cell strainer to produce single-cell suspensions. Dissociated cells were washed twice in PBS and incubated with DAPI (blue). For analysis, a typical forward- and side-scatter gate was set to exclude dead cells and aggregates; a total of 2 × 106 events in the gate were collected using the BD Biosciences LSR Fortessa cytometer. tdTomato positive cells were quantified using Flowjo software.

### Kaplan–Meier analysis

Kaplan–Meier plots were generated through the Jetset best probes (dataset number # ‘35617_at’ for ERK5 and ‘208820_at’ for FAK) to evaluate the prognostic significance of *ERK5* and *FAK* mRNA in distant metastasis-free survival (http://www.kmplot.com). For Fig. 1, patient specimens were divided into high and low expression groups according to the median expression. The median was computed before any subgroups were analyzed. Consequently, the same threshold of *ERK5* transcript level was utilized in all panels. The grade subgroup information was determined in the website database. For Figs. 2B, S4A and S4C, patient data were analyzed using the best cutoff to distinguish high and low expression groups. Patients marked as ‘ER negative’, ‘PR negative’ and ‘HER2 negative’ were grouped as TNBC patients. Hazard ratios (HR) with 95% confidence intervals and logrank *P* values were calculated automatically by the website software.

### Statistical analyses

Results were analyzed using the two-way ANOVA test to compare time courses. For normally distributed data, an unpaired two-tailed Student’s *t* test was performed. Statistical significance was set at *p ≤ 0.05, **p ≤ 0.01, ***p ≤ 0.001; *ns* indicates no statistical difference. For in vivo analysis of metastatic burden, the number of breast cancer cells extracted from the lung of paired tumor-bearing mice was normalized as ratios ($\frac{{dox - }}{{dox + }}$, when dox+ is 0, calculate it as 1) and compared using a ratio paired *t* test.

Additional materials and methods are listed in the Supplementary information.

## Supplementary information

Supplementary Videos

Supplementary Information

## Data Availability

The authors declare that all the data supporting the findings of this study are available within the paper and its supplementary information files. All other data supporting the findings of this study are available from the corresponding authors upon reasonable request.
