# Supplementary material for: The extracellular-regulated protein kinase 5 (ERK5) enhances metastatic burden in triple-negative breast cancer through focal adhesion protein kinase (FAK)-mediated regulation of cell adhesion
Source: Oncogene. 2021 May 12;40(23):3929–41. doi: 10.1038/s41388-021-01798-2 (PMC8195737; doi:10.1038/s41388-021-01798-2)
Supplement: Supplementary file 1 — Supplementary Videos [file 41388_2021_1798_MOESM1_ESM.pptx]

## Slide 1
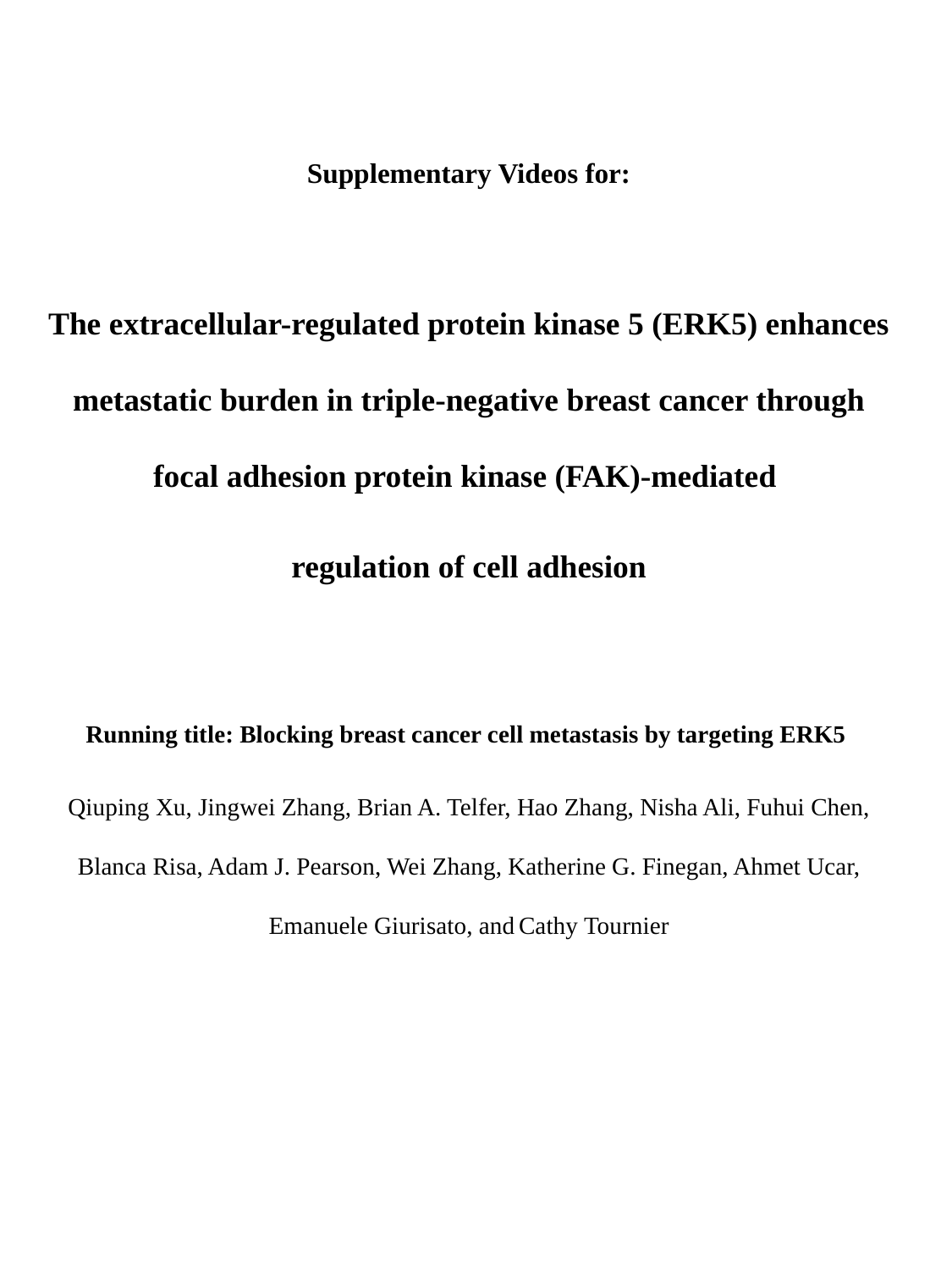

Supplementary Videos for:
The extracellular-regulated protein kinase 5 (ERK5) enhances metastatic burden in triple-negative breast cancer through focal adhesion protein kinase (FAK)-mediated
regulation of cell adhesion
Running title: Blocking breast cancer cell metastasis by targeting ERK5
Qiuping Xu, Jingwei Zhang, Brian A. Telfer, Hao Zhang, Nisha Ali, Fuhui Chen, Blanca Risa, Adam J. Pearson, Wei Zhang, Katherine G. Finegan, Ahmet Ucar, Emanuele Giurisato, and Cathy Tournier

## Slide 2
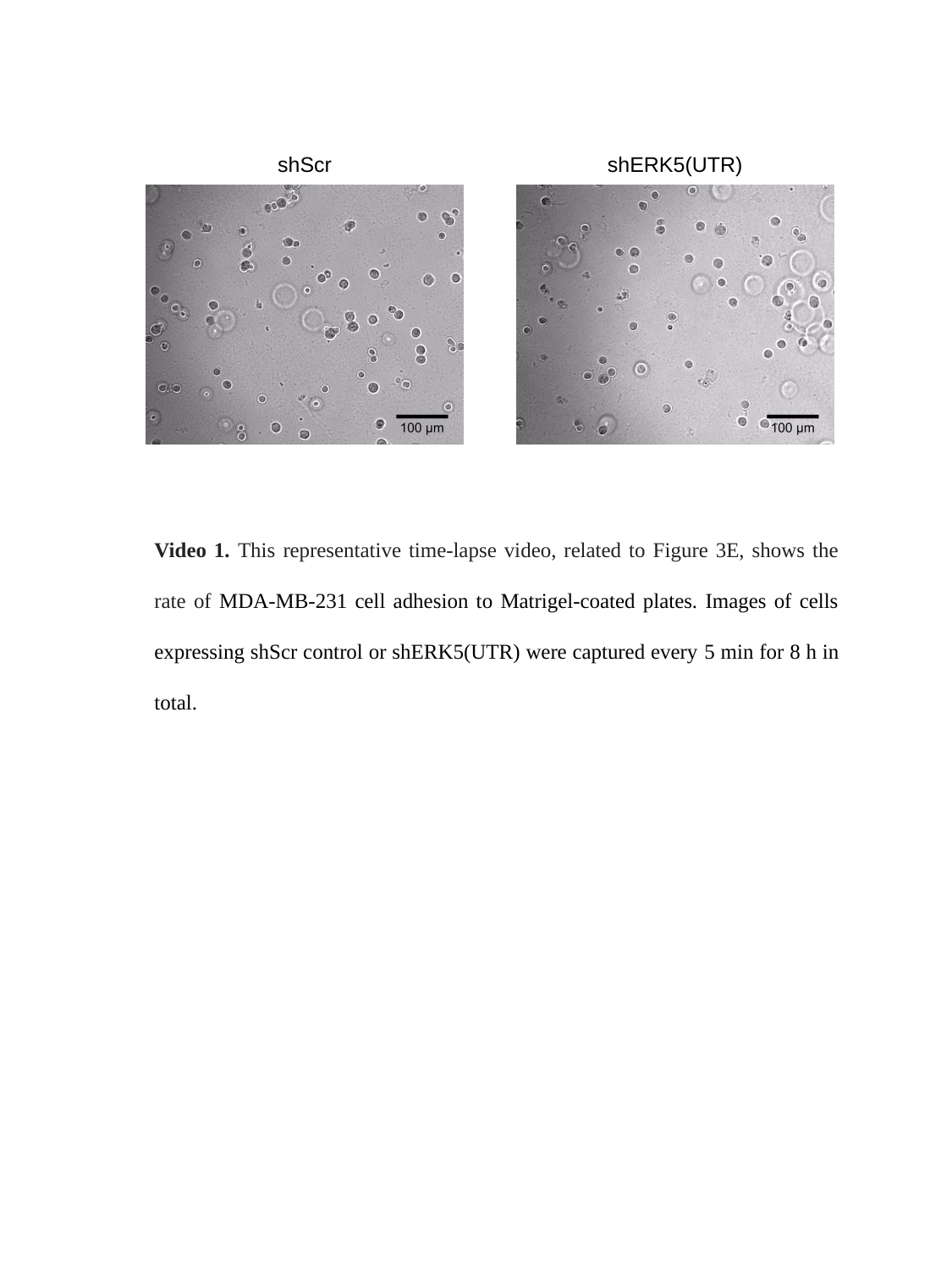

shScr
shERK5(UTR)
Video 1. This representative time-lapse video, related to Figure 3E, shows the rate of MDA-MB-231 cell adhesion to Matrigel-coated plates. Images of cells expressing shScr control or shERK5(UTR) were captured every 5 min for 8 h in total.

## Slide 3
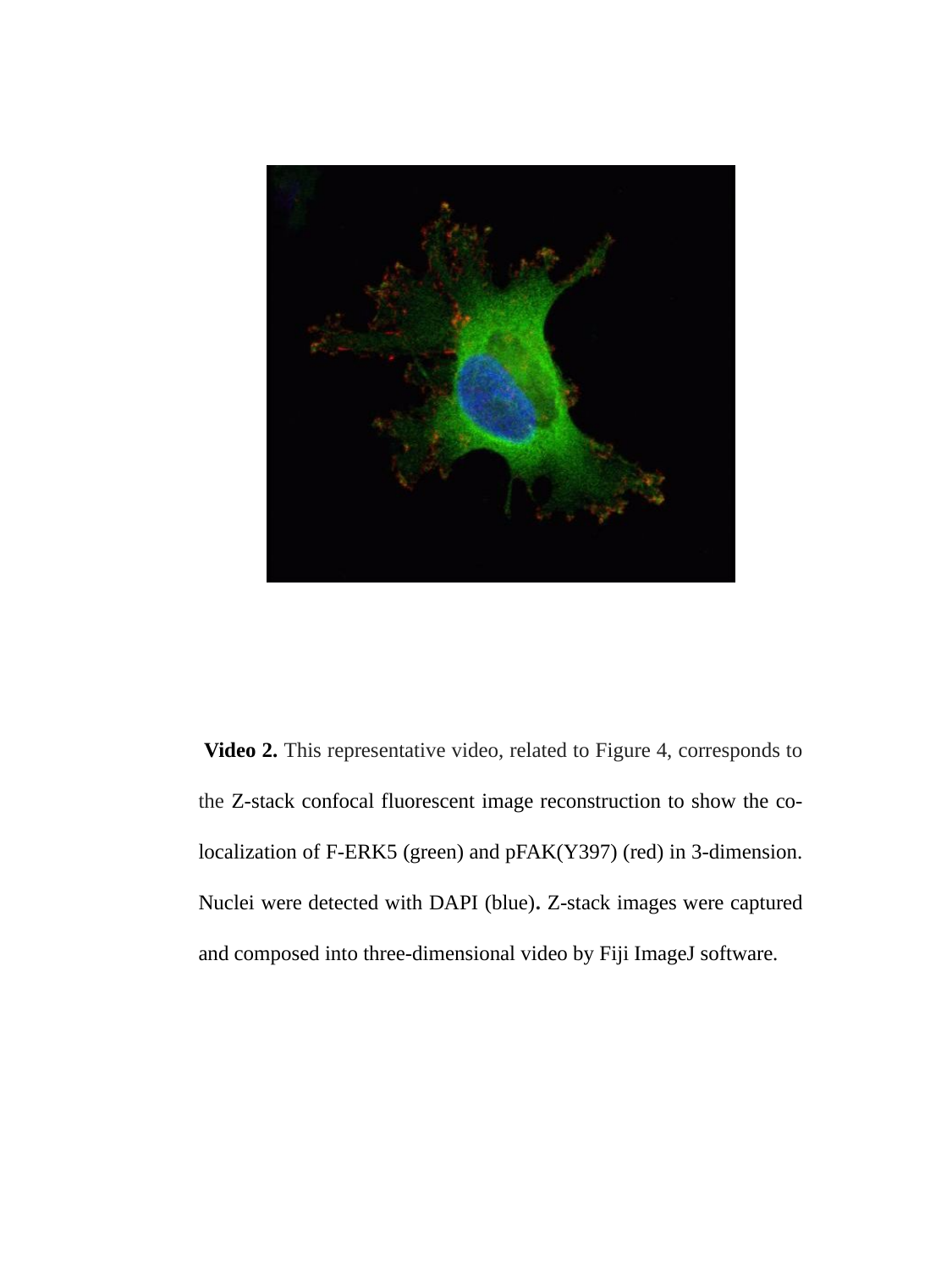

Video 2. This representative video, related to Figure 4, corresponds to the Z-stack confocal fluorescent image reconstruction to show the co-localization of F-ERK5 (green) and pFAK(Y397) (red) in 3-dimension. Nuclei were detected with DAPI (blue). Z-stack images were captured and composed into three-dimensional video by Fiji ImageJ software.
