# Supplementary material for: The extracellular-regulated protein kinase 5 (ERK5) enhances metastatic burden in triple-negative breast cancer through focal adhesion protein kinase (FAK)-mediated regulation of cell adhesion
Source: Oncogene. 2021 May 12;40(23):3929–41. doi: 10.1038/s41388-021-01798-2 (PMC8195737; doi:10.1038/s41388-021-01798-2)
Supplement: Supplementary file 2 — Supplementary Information [file 41388_2021_1798_MOESM2_ESM.pdf]

Supplementary Information for:

The extracellular-regulated protein kinase 5 (ERK5) enhances metastatic burden in triple-negative breast cancer through focal adhesion protein kinase (FAK)-mediated regulation of cell adhesion

Running title: Blocking breast cancer cell metastasis by targeting ERK5

Qiuping Xu, Jingwei Zhang, Brian A. Telfer, Hao Zhang, Nisha Ali, Fuhui Chen, Blanca Risa, Adam J. Pearson, Wei Zhang, Katherine G. Finegan, Ahmet Ucar, Emanuele Giurisato, and Cathy Tournier

Supplementary Materials and Methods

Table S1

Figures S1 to S7

Supplementary Materials and Methods

Materials

MDA-MB-231 and HEK293T cell lines were purchase from ATCC. Luciferin was purchased from Promega. Dulbecco's Modified Eagle's Medium (DMEM), Tryspin-EDTA, penicillin and streptomycin, doxycycline, puromycin, and MTT were purchased from Sigma-Aldrich. FBS utilized for culturing MDA-MBA-231 cells and HEK293 cells was from Sigma-Aldrich and Gibco, respectively. Tet-approved FBS utilized for doxycycline treatment of MDA-MB-231 cells was purchased from TAKARA. Matrigel was purchased from Corning. DAPI was from Sigma-Aldrich.

Animal welfare and human tissue sample protocols

Mice were maintained in a pathogen-free facility at the University of Manchester. All animal procedures were performed under license in accordance with the UK Home Office Animals (Scientific Procedures) Act (1986) and approved by the Animal Welfare and Ethical Review Body of the University of Manchester, UK. In particular, mice with tumors were closely monitored daily for any changes in their overall conditions. Formalin-fixed paraffin-embedded (FFPE) human breast tissues were obtained with appropriate ethical consents from the Manchester Cancer Research Centre (MCRC) Biobank, UK. The role of the MCRC Biobank is to distribute research samples and therefore, cannot endorse studies performed or the interpretation of results.

Cell culture and treatments

MDA-MB-231 and HEK293T cells were cultured in DMEM containing 10% FBS in the presence of antibiotics in a humidified 5% CO_2 incubator at 37°C. Where indicated, MDA-MB-231 cells were treated with 100 nM PND1186 (a gift from Andrew Gilmore, University of Manchester) to inhibit FAK, or with doxycycline (10 ng/ml or 2 μg/ml) for induced expression systems.

Virus production

Lentiviruses were generated by co-transfecting HEK293T cells with pLKO or pCHD recombinant plasmids with the psPAX2 packaging plasmid (Addgene #12260) and the pMD2.G envelop plasmid (Addgene #12259), as previously described (1, 2). The medium was replaced after 24 h. Supernatants containing lentiviral particles were collected 72 h after transfection, filtered through 0.22 µm filters and concentrated using the PEG-it reagent (System Biosciences #LV825A-1).

Immunoblot analysis

Proteins were extracted from cells in RIPA buffer (Sigma-Aldrich # R0278) containing protease and protein phosphatase inhibitors. Extracts (25 μg) were resolved by SDS-polyacrylamide gel electrophoresis (SDS-PAGE) and subjected to immunoblot analysis with the following antibodies from Cell Signaling Technology, unless indicated otherwise: ERK5 (#3372), p-FAK(Y397) (#3283), p-FAK(S910) (ThermoFisher Sci #44-596G), FAK (#3285), E-cadherin (Abcam #ab40772), p-ERK1/2 (#4370), or β-tubulin (#5346). Immunecomplexes

were detected by enhanced chemiluminescence with immunoglobulin G coupled to horseradish peroxidase as the secondary antibody (GE Healthcare #NA931 and #NA934).

Immunohistochemical analyses of human breast tissues and mammary tumor grafts.

For histological analysis, sections were stained with hematoxylin and eosin (H&E). For immunohistochemistry, paraffin embedded sections were incubated with antibodies to ERK5 (1:100 dilution for human sections and 1:1000 for mouse sections; Abcam #ab196609) or to p-FAK(Y397) (1:200 dilution; ThermoFisher #44-624G). Antigens were revealed using diaminobenzidine (DAB, brown) peroxidase substrate kit (Vector Laboratories #SK-4100) and counter stained with haematoxylin (blue), as described previously (3).

Confocal microscopy

MDA-MB-231 cells seeded in Matrigel were fixed in 2% paraformaldehyde and permeabilized in IF buffer (97.7 mM NaN3, 0.1% bovine serum albumin, 0.2% Triton X-100, 0.05% Tween-20 in PBS) (4), prior to being incubated with specific antibodies (1:500 dilution) to the FLAG epitope (Sigma-Aldrich #F1804) or to phosphorylated FAK at tyrosine 397 (ThermoFisher #44-624G). Immune complexes were detected using a secondary antibody (1:1000 dilution) conjugated to Alexa Fluor 488 or 594 (ThermoFisher #A32723 and #A32740). Nuclei were stained with DAPI (blue). Fluorescent images were viewed using a Leica SP8 confocal microscope.

Quantitative real-time PCR

Total RNA was reversed transcribed to cDNA using SuperScript™ II Reverse Transcriptase (Thermofisher Cat#18064022) and quantified by RT-PCR using the Power SYBR™ Green PCR Master Mix (Thermofisher Cat#4368706). Sequences of the forward and reverse primers are indicated as followed: *E-cad*-forward 5' GAAAGCGGCTGATACTGACC and *E-cad*-reverse 5'CGTACATGTCAGCCGCTTC; *Slug*-forward 5'GTGATTATTTCCCCGTATCTCT AT and *Slug*-reverse 5'CAATGGCATGGGGGTCTGAAAG; *Snail*-forward 5'GCTGCCAAT GCTCATCTGGGACTCT and *Snail*-reverse 5'TTGAAGGGCTTTCGAGCCTGGAGAT; *N-cad*-forward 5'TGTTTGACTATGAAGGCAGTGG and *N-cad*-reverse 5'TCAGTCATCACC TCCACCAT; *MMP2*-forward 5'ATGCCGCCTTTAACTGGAG and MMP2-reverse 5'GGAA AGCCAGGATCCATTTT; *Vimentin*-forward 5'GTCCACTGAGTACCGGAGACA and *Vimentin*-reverse 5'TAGTTAGCAGCTTCAACGGCAA; *RPLP0*-forward 5' GGCGACCTGG AAGTCCAA and *RPLP0*-reverse 5'CCATCAGCACCACAGCCTT. Results were analyzed using the $2^{-\Delta\Delta G}$ method. The level of expression of mRNA was normalized to *Rplp0*.

References

1. Wiederschain D, Wee S, Chen L, Loo A, Yang G, Huang A, Chen Y, Caponigro G, Yao YM, Lengauer C, Sellers WR, Benson JD. Single-vector inducible lentiviral RNAi system for oncology target validation. Cell Cycle. 2009;8:498-504.

2. Wee S, Wiederschain D, Maira S-M, Loo A, Miller C, deBeaumont R, et al. PTEN-deficient cancers depend on PIK3CB. Proc Natl Acad Sci U S A. 2008;105:13057-62.

3. Giurisato E, Xu Q, Lonardi S, Telfer B, Russo I, Pearson A, et al. Myeloid ERK5 deficiency suppresses tumor growth by blocking protumor macrophage polarization via STAT3 inhibition. Proc Natl Acad Sci U S A. 2018;115:E2801-E2810.

1. Ran R, Harrison H, Syamimi Ariffin N, Ayub R, Pegg HJ, Deng W, et al. A role for CBFβ in maintaining the metastatic phenotype of breast cancer cells. Oncogene. 2020;39:2624-37.

Supplementary Table S1

Oligonucleotides used in this study

	**Oligos for constructing Tet-pLKO-shERK5i**
Forward	CCGG**GACCCACCTTTCAGCCTTA**CTCGAG**TAAGGCTGAAAGGTGGGTC**TTTTT
Reverse	AATTAAAAA**GACCCACCTTTCAGCCTTA**CTCGAG**TAAGGCTGAAAGGTGGGTC**
	**Primers to amplify F-ERK5(WT)**
Forward	GAGATTAATTAACCACCATGGACTACAAAGACCAT
Reverse	GAGAGCTAGCTCAGGGGTCCTGGAG
	**Primers to mutate ERK5(D200A)**
Forward	CACGAGCCATACCAAAGGCACCAATCTTGAGCTCA
Reverse	TGAGCTCAAGATTGGTGCCTTTGGTATGGCTCGTG

Supplementary Figure legends

Figure S1: ERK5 is overexpressed in triple negative breast cancer. Biopsies of human invasive triple-negative ductal carcinoma and adjacent normal breast tissues were stained with H&E or with a specific antibody to ERK5 (brown). The red stars indicate cancerous cells exhibiting nuclear ERK5 staining in the biopsy of patient #750. Interestingly, this patient originally received a pre-surgery diagnosis of ER+ tumor, but was later confirmed as TNBC subtype from the histopathology report of the biopsy. Two TNBC patients (1829 and 2889) exhibiting distant metastasis in the left upper or lower lobe (pulmonary nodules 1 and 2) and right upper lobe (pulmonary nodule 3) of the lung were included**.** Scale bars: (5x) 500 µm, (10x) 100 µm, (40x) 20 µm.

Figure S2: ERK5 is required for TNBC cell invasion. (**A**) Immunoblot analysis of ERK5 expression in human luminal, HER2-expressing (+) and triple negative (TN) breast cancer cell lines. β-actin was utilized as loading control. Similar results were obtained in two independent experiments. (**B**) Effect of ERK5 knockdown on the growth of MDA-MB-231 cells by 3-(4,5-dimethylthiazol-2-yl)-2,5-diphenyltetrazolium bromide (MTT) assay. 5,000 cells/well were plated in 96-well plates and cultured in FBS-containing medium for the indicated times. Optical densities were determined at 570 nm. The data correspond to the mean ± SD (N=2). (**C**) Effect of ERK5 knockdown on MDA-MB-231 cell invasion. The cells were re-suspended in serum-free medium in Boyden chambers coated with Matrigel. The lower chamber was filled with FBS-free media (basal), or with media containing 10% FBS (serum) or 20 ng/mL EGF. The data correspond to the mean ± SD (N=3).

Figure S3: ERK5 promotes breast cancer cell invasion through mediating FAK activation. (**A)** iRFP720$^+$ MDA-MB-231 cells carrying shERK5(UTR) were incubated for 24 h with 2 μg/ml doxycycline to induce F-ERK5-ΔC(1-575) expression. Cells were subsequently seeded on Matrigel-coated glass bottom plate for 6 h prior to being fixed and incubated with antibodies to the FLAG epitope (M2) and p-FAK(Y397). Negative controls included incubation with each primary antibody separately and no primary antibodies. *In situ* detection of ERK5ΔC-FAK complexes was performed with oligonucleotide-labeled secondary antibodies according to the Duolink® PLA fluorescence protocol (Sigma). Images were acquired by fluorescence microscopy. Scale bars: 10 μm. (**B**) MDA-MB-231 cells were starved overnight prior to being stimulated with EGF (50 ng/mL) for 30 minutes. Where indicated the cells were pre-incubated with 2 μM JWG-045. Immunoblot analysis of the cell lysates confirmed that the slow migrating band detected in EGF-treated MDA-MB-231 cell extracts disappeared as a consequence of inhibition of ERK5 activity by JWG-045 and the loss of C terminal tail autophosphorylation. (**C**) MDA-MB-231 cells were incubated with DMSO or with 2 μM JWG-045 for 1 h prior to being seeded to Matrigel-coated plates and cultured for 12 h in FBS-free media containing DMSO or JWG-045, respectively. Cell lysates were analyzed by immunoblot alongside samples obtained from MDA-MB-231 cell expressing shScr or shERK5(UTR). β-tubulin was utilized as a loading control. (**D**) iRFP720$^+$ cells expressing shERK5(UTR) were incubated with 10 ng/ml doxycycline for 24 h to induce F-ERK5(WT) or F-ERK5(D200A) expression, prior to being seeded in FBS-free Matrigel-coated plates and cultured for 12 h. Alternatively, MDA-MB-231 cells expressing shERK5(UTR) were transfected with a plasmid expressing (myr)FAK for 24 h. MDA-MB-231 cells expressing shScr control or transduced with an empty pCHD plasmid (-) were utilized as controls. Lysates were analysed by immunoblot. Similar results were obtained in two independent experiments.

(**E**) The cells were seeded in Matrigel coated Boyden chamber and analyzed by invasion assays. The bar graph indicates the mean ± SD (N=3).

Figure S4: Pharmacological inhibition of FAK reproduces the invasion defect phenotype caused by silencing ERK5.

(**A**) The level of FAK positively correlates with increased risk of breast cancer metastasis. The relationship between the level of FAK expression and distant metastasis-free survival of all breast cancer (BC) patients was assessed by Kaplan-Meier plotter. The graph shows that patients scored as being “high FAK” (red) developed metastasis earlier compared with those exhibiting “low FAK” (black). Hazard ratio (HR) and logrank P values are shown. (**B**) The level of *Fak* transcript was analyzed in 416 luminal/HER2+ tumors and 98 TNBC from TCGA dataset. Black lines in each group indicate median with interquartile range. The analysis shows that *Fak* transcript level was the highest in basal-like tumors among all subtypes. (**C**) Kaplan-Meier analysis of distant metastasis-free survival of TNBC patients. Samples were divided as in (A). The data indicate that level of FAK expression is predictive of TNBC metastasis (hazard ratio = 4), although the small size of the cohort meant that this analysis did not reach statistical significance (p value = 0.2; Fig. 6C). (**D**) FAK inhibition block MDA-MB-231 cell invasion. The cells were pre-incubated for 1 h with 100 nM PND1186 prior to being seeded in Boyden chambers coated with Matrigel. PND1186 was added in the lower chamber. The data correspond to the mean ± SD (N=3). Representative pictures of cells seeded in Matrigel-coated plates and cultured for 4 h in serum-free medium containing DMSO or 100 nM PND1186. (**E**) MDA-MB-231 cells growing in FBS-free medium on Matrigel-coated plates were incubated with 100 nM PND1186 for 4 h. The effect of PND1186 treatment on FAK, ERK5 and E-cadherin (E-cad) expression and on the phosphorylation of FAK and ERK1/2 was examined

by immunoblot analysis. β-tubulin were used as loading control. Similar results were obtained in two independent experiments.

Figure S5: Strategy for inducible ERK5 knockdown *in vivo*. (**A**) Schematic representation of the strategy utilized for doxycyclin-induced ERK5 silencing in MDA-231-MB cells. A modified polycistronic lentiviral transfer plasmid containing shERK5i under the control of a Tet-on promoter and Luc2=tdT driven by the hPGK promoter was engineered and packaged into viral particles. Transduced tdT positive MDA-MB-231 cells were selected by incubation with puromycin for stable integration of shERK5i. Immunoblot analysis confirmed efficient ERK5 knockdown in recombinant MDA-MB-231 cells incubated with 2 µg/ml doxycycline (+) for 24 h. Mock treated cells with DMSO (-) were used as controls. (**B**) Mass spectrometry analyse of murine plasma. The graphs show a much more concentrated level of doxycycline in the plasma of mice exposed to a doxycycline-containing diet (number 2, 4, 6) compared with mice fed with a standard diet (number 1, 3, 5). Water and doxycycline-diluted samples was used as standard (top panel).

Figure S6: Induced ERK5 knockdown enhances the growth of breast tumor graft. 2×10^6 MDA-MB-231 cells carrying shERK5i were subcutaneously implanted into the back of CBA nude mice. The following day, the mice were fed with a doxycycline containing diet (dox+) to silence ERK5 in breast cancer cells or with a standard doxycycline-free (dox-) diet to produce control animals. (**A**) Representative bioluminescence images and pictures of mammary tumor grafts excised from sacrificed animals 56 days after implantation. (**B**) The growth of tumor grafts was monitored over time after initial cell injection. The data presented as fold increase

in tumor size after the initial graft was established at day 43 correspond to the mean ± SD. Total N number of animals per condition is indicated.

Figure S7: Detection of FAK phosphorylation at Tyr397 in human invasive ductal carcinoma correlates with high ERK5 expression. Serial sections of human invasive ductal carcinoma were stained with H&E or with specific antibodies (brown) to p-FAK(Y397) or to ERK5. Scale bars: (5x) 200 µm, (20x) 50 µm, (50x) 20 µm.

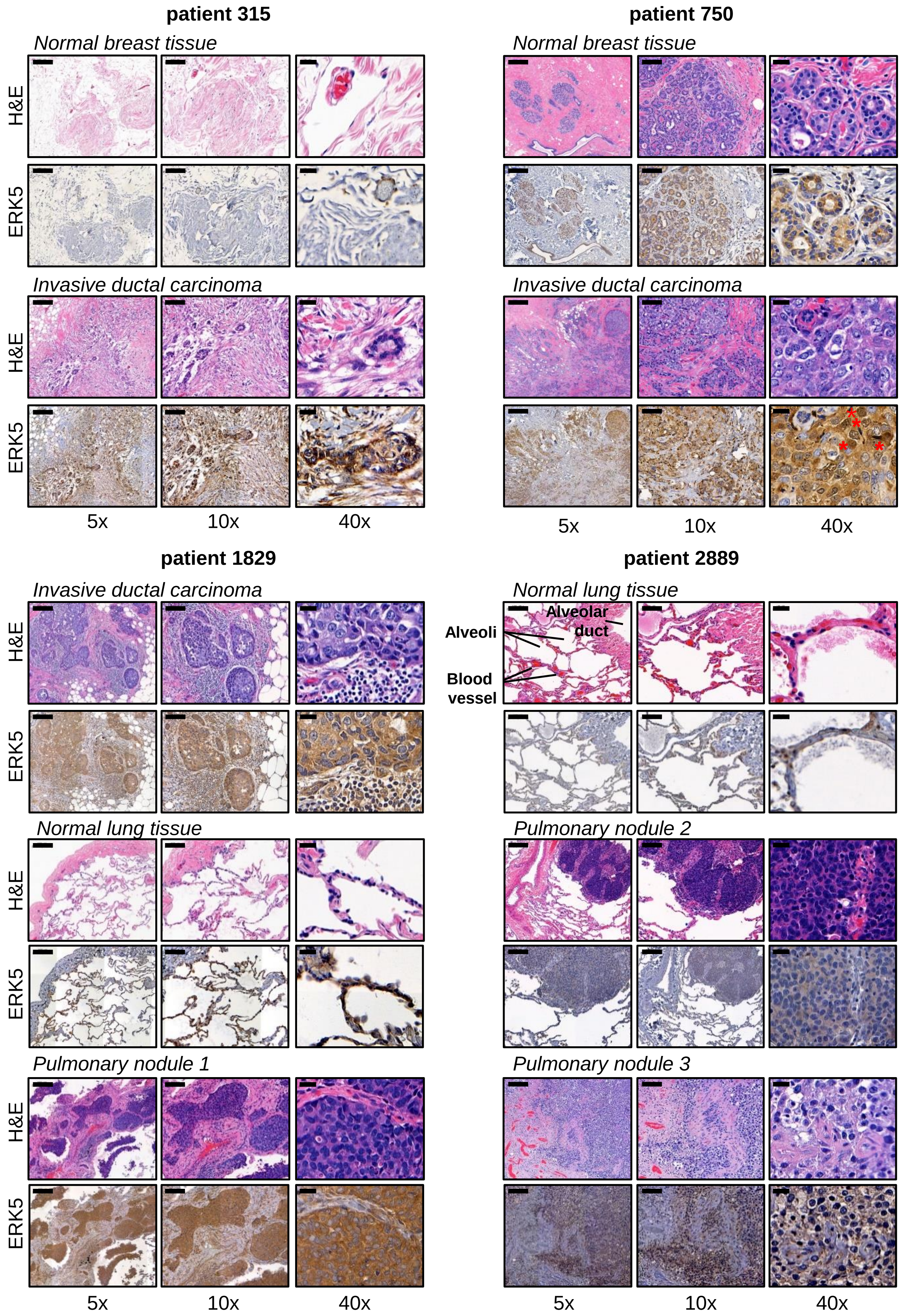

patient 315
Normal breast tissue
H&E
ERK5
Invasive ductal carcinoma
H&E
ERK5
5x
10x
40x
patient 750
Normal breast tissue
Invasive ductal carcinoma
5x
10x
40x
patient 1829
Invasive ductal carcinoma
H&E
ERK5
Normal lung tissue
H&E
ERK5
Pulmonary nodule 1
H&E
ERK5
5x
10x
40x
patient 2889
Normal lung tissue
Alveolar duct
Alveoli
Blood vessel
Pulmonary nodule 2
Pulmonary nodule 3
5x
10x
40x

Figure S1

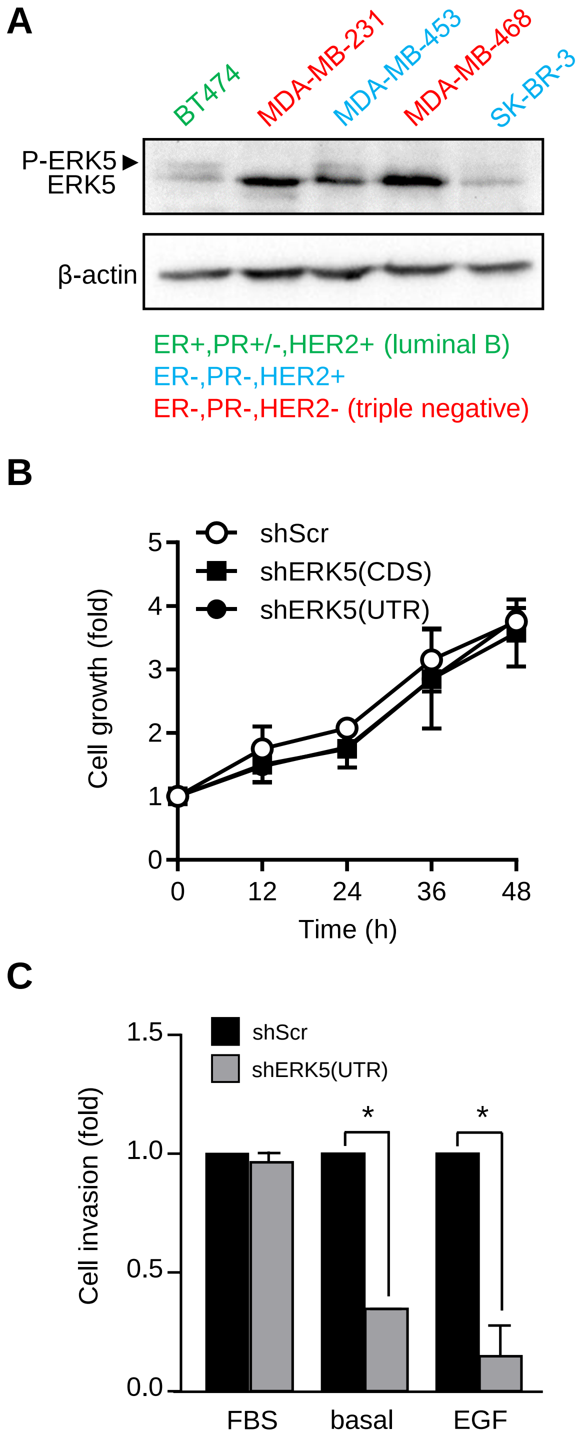
A
BT474
MDA-MB-231
MDA-MB-453
MDA-MB-468
SK-BR-3
P-ERK5
ERK5
β-actin
ER+,PR+/-,HER2+ (luminal B)
ER-,PR-,HER2+
ER-,PR-,HER2- (triple negative)
B
shScr
shERK5(CDS)
shERK5(UTR)
Cell growth (fold)
0
1
2
3
4
5
0
12
24
36
48
Time (h)
C
shScr
shERK5(UTR)
Cell invasion (fold)
1.5
1.0
0.5
0.0
*
*
FBS
basal
EGF

Figure S2

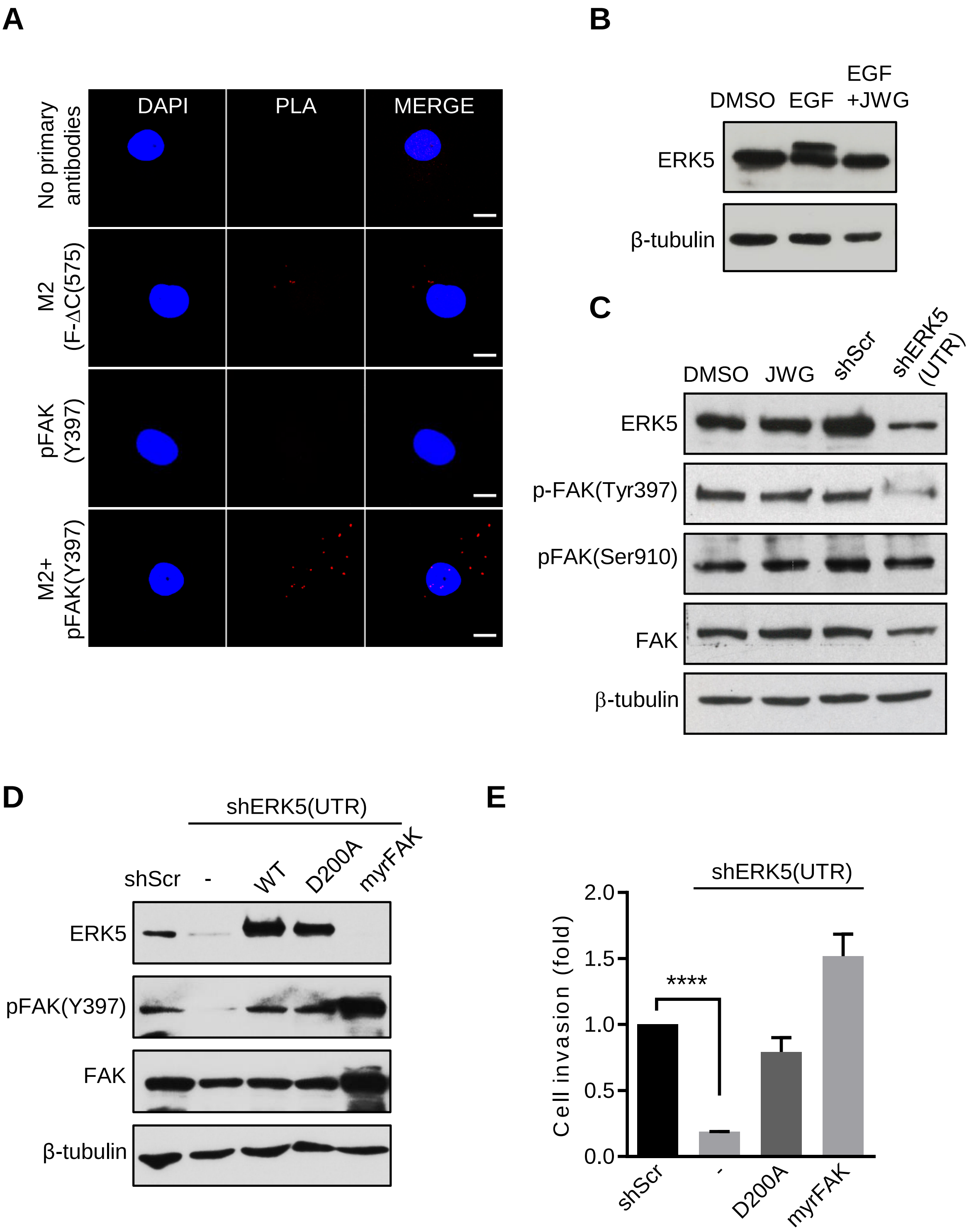
A
DAPI
PLA
MERGE
No primary antibodies
M2 (F-ΔC(575))
pFAK (Y397)
M2+ pFAK(Y397)
B
DMSO
EGF
EGF +JWG
ERK5
β-tubulin
C
DMSO
JWG
shScr
shERK5 (UTR)
ERK5
p-FAK(Tyr397)
pFAK(Ser910)
FAK
β-tubulin
D
shERK5(UTR)
shScr
-
WT
D200A
myrFAK
ERK5
pFAK(Y397)
FAK
β-tubulin
E
shERK5(UTR)
Cell invasion (fold)
2.0
1.5
1.0
0.5
0.0
****
shScr
-
D200A
myrFAK

Figure S3

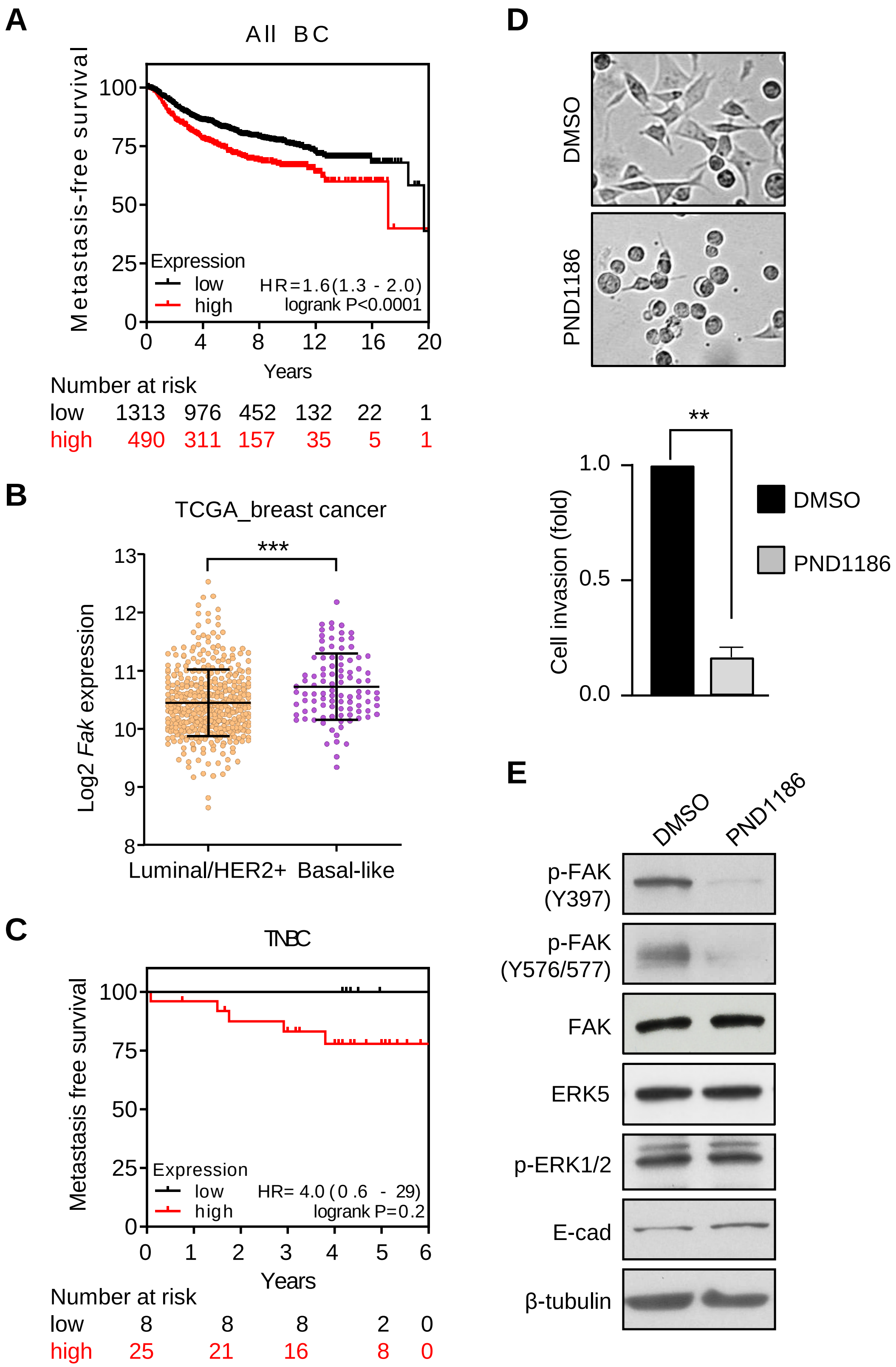
A
All BC
Metastasis-free survival
100
75
50
25
0
Expression
low
high
HR=1.6(1.3 - 2.0)
logrank P<0.0001
0 4 8 12 16 20
Years
Number at risk
low 1313 976 452 132 22 1
high 490 311 157 35 5 1
B
TCGA_breast cancer
***
Log2 Fak expression
13 12 11 10 9 8
Luminal/HER2+ Basal-like
C
TNBC
Metastasis free survival
100
75
50
25
0
Expression
low
high
HR= 4.0 (0 .6 - 29)
logrank P=0.2
0 1 2 3 4 5 6
Years
Number at risk
low 8 8 8 2 0
high 25 21 16 8 0
D
DMSO
PND1186
**
Cell invasion (fold)
1.0
0.5
0.0
DMSO
PND1186
E
DMSO
PND1186
p-FAK (Y397)
p-FAK (Y576/577)
FAK
ERK5
p-ERK1/2
E-cad
β-tubulin

Figure S4

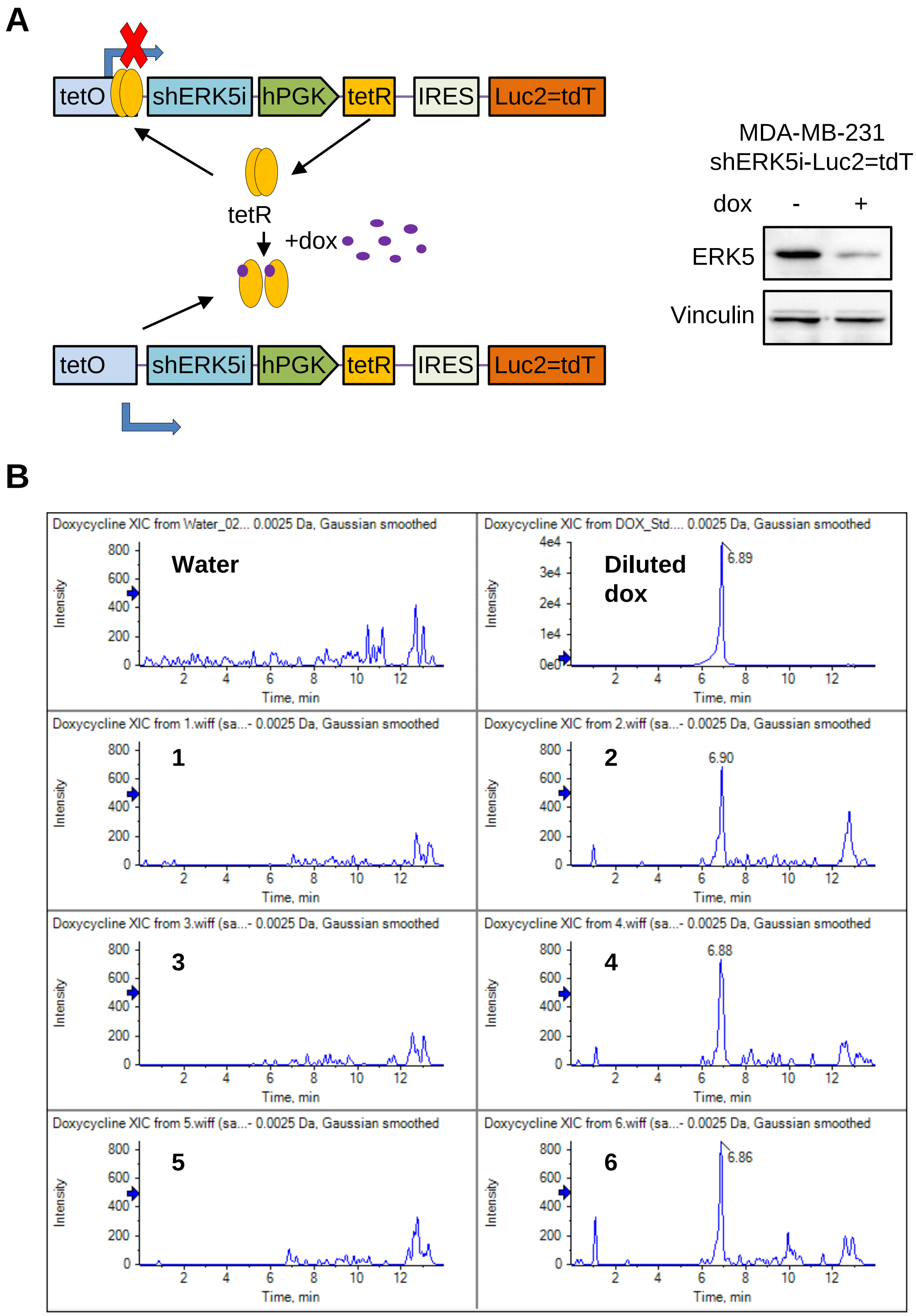
A
tetO
shERK5i
hPGK
tetR
IRES
Luc2=tdT
tetR
+dox
MDA-MB-231
shERK5i-Luc2=tdT
dox - +
ERK5
Vinculin
B
Doxycycline XIC from Water_02... 0.0025 Da, Gaussian smoothed
Water
Doxycycline XIC from DOX_Std.... 0.0025 Da, Gaussian smoothed
Diluted dox
6.89
Doxycycline XIC from 1.wiff (sa...- 0.0025 Da, Gaussian smoothed
1
Doxycycline XIC from 2.wiff (sa...- 0.0025 Da, Gaussian smoothed
2
6.90
Doxycycline XIC from 3.wiff (sa...- 0.0025 Da, Gaussian smoothed
3
Doxycycline XIC from 4.wiff (sa...- 0.0025 Da, Gaussian smoothed
4
6.88
Doxycycline XIC from 5.wiff (sa...- 0.0025 Da, Gaussian smoothed
5
Doxycycline XIC from 6.wiff (sa...- 0.0025 Da, Gaussian smoothed
6
6.86
Intensity
Time, min

Figure S5

A

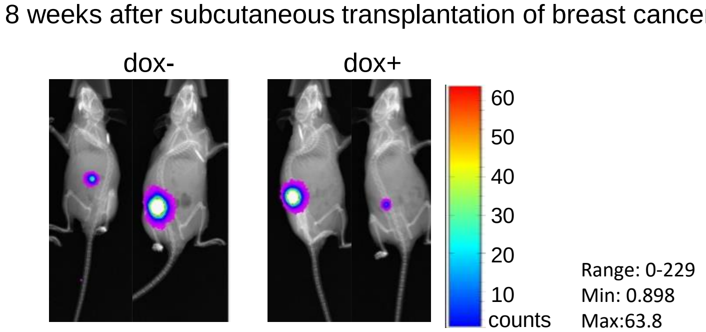
8 weeks after subcutaneous transplantation of breast cancer cells
dox-
dox+
60
50
40
30
20
10
counts
Range: 0-229
Min: 0.898
Max:63.8

B

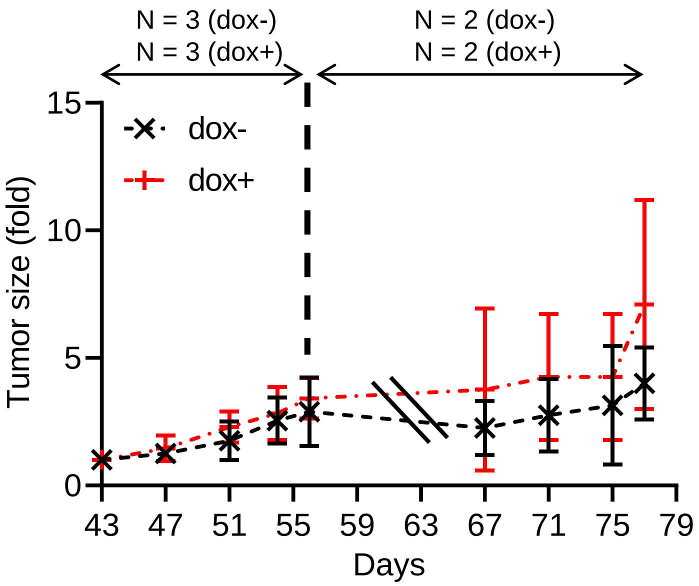
N = 3 (dox-)
N = 3 (dox+)
N = 2 (dox-)
N = 2 (dox+)
dox-
dox+
Tumor size (fold)
15
10
5
0
43
47
51
55
59
63
67
71
75
79
Days

Figure S6

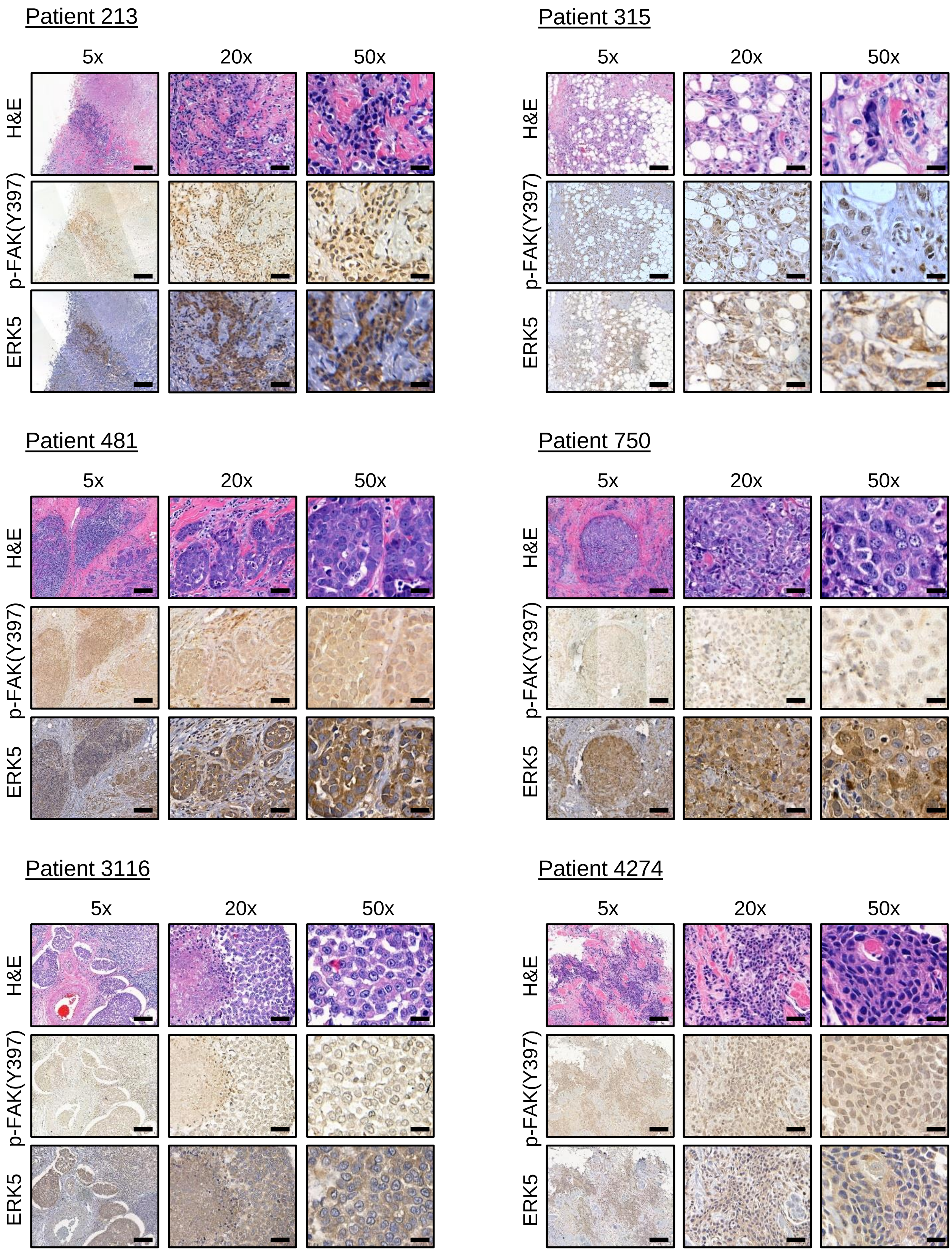
Patient 213
5x
20x
50x
H&E
p-FAK(Y397)
ERK5
Patient 315
5x
20x
50x
H&E
p-FAK(Y397)
ERK5
Patient 481
5x
20x
50x
H&E
p-FAK(Y397)
ERK5
Patient 750
5x
20x
50x
H&E
p-FAK(Y397)
ERK5
Patient 3116
5x
20x
50x
H&E
p-FAK(Y397)
ERK5
Patient 4274
5x
20x
50x
H&E
p-FAK(Y397)
ERK5

Figure S7
